# How to incorporate patient and public perspectives into the design and conduct of research

**DOI:** 10.12688/f1000research.15162.1

**Published:** 2018-06-18

**Authors:** Pat Hoddinott, Alex Pollock, Alicia O'Cathain, Isabel Boyer, Jane Taylor, Chris MacDonald, Sandy Oliver, Jenny L. Donovan

**Affiliations:** 1 Nursing, Midwifery and Allied Health Professions Research Unit, Faculty of Health Sciences and Sport, University of Stirling, Stirling, FK9 4LA, UK; 2 Nursing, Midwifery and Allied Health Professions Research Unit, Glasgow Caledonian University, Glasgow, G4 0BA, UK; 3 Medical Care Research Unit, School of Health and Related Research, University of Sheffield, Sheffield, S1 4DA, UK; 4 PPI member of NIHR/HTA General Board, NIHR Evaluation, Trials and Studies Coordinating Centre, Southampton, SO16 7NS, UK; 5 Chair of Patient Insight Group, Arthritis Research UK, Chesterfield, S41 7TD, UK; 6 Research Involvement Manager, Arthritis Research UK, Chesterfield, S41 7TD, UK; 7 Department of Social Science, Social Science Research Unit, UCL Institute of Education, University College London, London, WC1H 0AL, UK; 8 School of Social and Community Medicin, University of Bristol, Bristol, BS8 2PS, UK; 9 NIHR Collaboration for Leadership in Applied Health Research and Care West (CLAHRC West), University Hospitals Bristol NHS Foundation Trust, Bristol, BS2 8HW, UK

**Keywords:** Public and Patient Involvement, Public Engagement, Qualitative research, Research Methods, Co-production, Partnership approaches

## Abstract

International government guidance recommends patient and public involvement (PPI) to improve the relevance and quality of research.  PPI is defined as research being carried out ‘with’ or ‘by’ patients and members of the public rather than ‘to’, ‘about’ or ‘for’ them ( http://www.invo.org.uk/). Patient involvement is different from collecting data from patients as participants.  Ethical considerations also differ.  PPI is about patients actively contributing through discussion to decisions about research design, acceptability, relevance, conduct and governance from study conception to dissemination.  Occasionally patients lead or do research.  The research methods of PPI range from informal discussions to partnership research approaches such as action research, co-production and co-learning.

This article discusses how researchers can involve patients when they are applying for research funding and considers some opportunities and pitfalls.  It reviews research funder requirements, draws on the literature and our collective experiences as clinicians, patients, academics and members of UK funding panels.

## Introduction

Patient and public involvement (PPI) is recommended from the earliest research stages through to dissemination of the findings ^1– 6^. In the UK, INVOLVE ^3^ states that research should be done with and by patients, but what does this mean for researchers and patient partners when starting a study? International resources are available ( Box 1) and six UK PPI standards are being tested to see if they work in practice ^7^. Table 1 summarises on-line guidance for research applications to international government funding programmes that endorse involving patients and the public. Language varies internationally and is evolving as patients take a more central role in deciding what research is done and how. Box 2 provides some definitions which are derived from the INVOLVE jargon buster ^8^ and international resources ( Table 1). PPI includes patients, potential patients, families, carers, patient groups and members of the public who use or have access to health and social care services ^3^. We refer to this broad group as ‘patients’ to distinguish them from clinicians and academics. This is consistent with Canadian guidance, which defines ‘patients’ as ‘an overarching term inclusive of individuals with personal experience of a health issue and informal caregivers, including family and friends’ ^9^. However, ‘patients’ may include people who do not describe themselves in this way. People may self-care for their condition and general public contributions can add value to research questions. Other relevant international terms, for example stakeholder involvement, consumer involvement, knowledge user engagement and patient orientated research are described in Supplementary File 1, Section A.

Box 1. **Useful resources for patient and public involvement (listed alphabetically).**

**International endorsement of public and patient involvement in research**

Australian Government National Health and Medical Research Council: Consumer and Community Involvement: https://www.nhmrc.gov.au/research/consumer-and-community-involvement

Australian Government National Health and Medical Research Council: Statement on Consumer and Community Participation in Health and Medical Research (the Statement on Participation): https://www.nhmrc.gov.au/guidelines-publications/r22

Canadian Institutes of Health Research, Strategy for Patient-Oriented Research (SPOR): A coalition dedicated to the integration of research into care: http://www.cihr-irsc.gc.ca/e/41204.html

Cochrane Consumer Network. Statement of Principles for Consumer Involvement in Cochrane: http://consumers.cochrane.org/news/statement-principles-consumer-involvement-cochrane

Cochrane Training. Involving People learning resource relating to systematic reviews, developed by the ACTIVE (Authors and Consumers Together Impacting on eVidencE) project: http://training.cochrane.org/ACTIVE

European Patient Academy (EUPATI) is a network of European National Platforms which supports the integration of patient involvement across the entire process of medicines research and provides training. This includes the pharmaceutical industry and regulatory agencies. https://www.eupati.eu/

European Health 2020 Strategy calls for civil society engagement to improve health: http://www.euro.who.int/en/publications/abstracts/health-2020-a-european-policy-framework-supporting-action-across-government-and-society-for-health-and-well-being

Gordon and Betty Moore Foundation and The American Institutes for Research: A roadmap for patient and family engagement in health and research: http://patientfamilyengagement.org/

Health Technology Assessment International Patient and Citizen Involvement: www.htai.org/interest-groups/patient-and-citizen-involvement/

Nuffield Council on Bioethics (2012) Emerging biotechnologies: technology, choice and the public good: http://nuffieldbioethics.org/project/emerging-biotechnologies/

Ottawa Charter for Health Promotion. World Health Organisation 1986: ( http://www.who.int/healthpromotion/conferences/previous/ottawa/en/index1.html)

Patient Centered Outcomes Research Institute (PCORI) standards: https://www.pcori.org/research-results/about-our-research/research-methodology

PCORI Engagement Rubric: https://www.pcori.org/sites/default/files/Engagement-Rubric.pdf

The European Group on Ethics in Science and New Technologies. (2015). Opinion on the ethical implications of new health technologies and citizen participation. Europa: https://ec.europa.eu/research/ege/pdf/opinion-29_ege_executive-summary-recommendations.pdf

US Department of Health and Human Services: Public Involvement with the National Institutes of Health: https://www.nih.gov/about-nih/what-we-do/get-involved-nih/public-involvement-nih

**Key UK-based resources and organisations**

Healthtalk.org. Patient and public involvement in research: personal stories of patient involvement in research: http://www.healthtalk.org/peoples-experiences/medical-research/patient-and-public-involvement-research/topics

INVOLVE: supports active public involvement in NHS, public and social care research. Funded by NIHR. http://www.invo.org.uk/. There are useful pages on ‘Budgeting for Involvement Guidance’: http://www.invo.org.uk/wp-content/uploads/2014/11/10002-INVOLVE-Budgeting-Tool-Publication-WEB.pdf and an Involvement Cost Calculator: http://www.invo.org.uk/resource-centre/payment-and-recognition-for-public-involvement/involvement-cost-calculator/

invoDIRECT is a directory of organisations, networks and groups that support active public involvement in research and helps people to identify activity in their area of interest. http://www.invo.org.uk/communities/invodirect/.

invoNET is a network of people who are building the evidence knowledge and learning about public involvement in research: http://www.invo.org.uk/communities/invonet/.

James Lind Alliance: bring patients, carers and clinicians together in Priority Setting Partnerships to identify and prioritise the top uncertainties, or unanswered questions, about the effects of treatments: http://www.jla.nihr.ac.uk/

National Co-ordinating Centre for Public Engagement (NCCPE) has sections for researchers (and others) to explore, support, plan and do public engagement. It runs training courses and helps Universities to engage with the public: https://www.publicengagement.ac.uk/

NHS Health Research Authority: protects and promotes the interests of patients and the public in health and social care research and has top tips on public involvement in grant applications: https://www.hra.nhs.uk/planning-and-improving-research/research-planning/public-involvement/

NICE’s approach to public involvement in guidance and standards: a practical guide (2015): https://www.nice.org.uk/media/default/About/NICE-Communities/Public-involvement/Public-involvement-programme/PIP-process-guide-apr-2015.pdf

NIHR Going the Extra Mile strategy: https://www.nihr.ac.uk/patients-and-public/documents/Going-the-Extra-Mile.pdf

NIHR Patient and Public involvement in research. https://www.nihr.ac.uk/patients-and-public/

NIHR Public involvement standards development: a project aiming to improve the quality and consistency of public involvement (PI) in research through the development and introduction of national standards: https://sites.google.com/nihr.ac.uk/pi-standards/home

NIHR Research design service: provides support to health and social care researchers across England on all aspects of developing a grant application including, research design, research methods, identifying funding sources and involving patients and the public. Their advice is confidential and free of charge: https://www.nihr.ac.uk/about-us/how-we-are-managed/our-structure/research/research-design-service/

Patients active in research: A website promoting partnership between patient, carers, members of the public and medical researchers, including case studies of patient involvement in research and opportunities to take part in medical research: https://patientsactiveinresearch.org.uk/

Patients included charters: provide entities with a means of demonstrating their commitment to incorporating the experience and insight of patients into their organisations by ensuring that they are neither excluded, nor exploited: https://patientsincluded.org/

People in research: helps researchers and research organisations to find patients to work with and advertises opportunities for public involvement in NHS, public health and social care research: https://www.peopleinresearch.org/

Research Councils UK Concordat for Engaging the Public with Research: https://www.ukri.org/public-engagement/

**Table 1.** International government research funding guidance that endorses patient and public involvement (accessed November 2017).

Government general research guidance ^*^	Terminology ^*^	Application form specific guidance	Key features of funding applications
Australian Government National Health and Medical Research Council (NHMRC): Guidelines on Consumer and Community Participation in Health and Medical Research endorse consumers right to make contributions	Consumer and Community Involvement Consumer and Community Participation	NHMRC advice and instructions to applicants: https://www.nhmrc.gov. au/book/nhmrc-advice-and-instructions-applicants-2017/nhmrc-advice- and-instructions-2017 Indigenous populations: https://www.nhmrc.gov.au/book/nhmrc-funding-rules/section-b-project- grants/b-attachment-d-guidance-applicants-address-criteri	Mandatory Plain English Summary. Consumer and community engagement sections are not always mandatory and applicants can decide ‘if applicable’. There is either a form section to complete which asks applicants to enter each community engagement activity separately or instructions to upload either a one or two page document. Fellowship applications require information about community engagement and participation for the previous 5 years or 10 years. Some forms have ‘if applicable’ sections which ask applicants to describe: a) how participants will have access to their own results and how the researchers will be accountable to participants for the overall results of the research: b) how they will ensure consumers are involved in the research and how they will communicate results to participants and the community.
Canadian Institutes of Health Research (CIHR), Strategy for Patient-Oriented Research (SPOR) Framework. Patient, Citizen and Knowledge User Engagement in research should be meaningful throughout the research process, to inform research planning and design.	Patient Orientated Research Patient engagement in research Citizen engagement in research Knowledge user engagement.	Current Opportunities: https://www.researchnet-recherchenet.ca Index of funding related forms: http://www.cihr-irsc.gc.ca/e/797.html Some application forms do not mention patient/citizen engagement, e.g. secondary analysis of existing datasets.	*SPOR funded opportunities* Mandatory research activities relating to the SPOR Framework, e.g. patients engaged as partners; focus on patients’ priorities; patients must play a key role in a multi-disciplinary team; a minimum of one Principal Applicant or Principal Knowledge User must be a patient; a workshop, roundtable, public lecture or citizen/patient engagement forum. A patient engagement plan is required Funding: applies a 1:1 matching formula with non-federal government partners. Applicants can request consultant fees for patient engagement experts and costs to facilitate engagement such as compensation, incentives, and the development of orientation and training. *Non SPOR funding opportunities* A lay summary is not always required. Some forms ask applicants to provide a Knowledge/ Technology Users (KTU) plan with activities that will translate the research results outside the academic environment. Funding may be available for indigenous populations. ‘Costs related to community mobilisation and engagement, including culturally relevant promotional items such as, tobacco, cloth, and cash reimbursements (in a method acceptable to the individual or community being reimbursed) to compensate community participation; and contracts and/or consultant fees for knowledge translation and communication activities for Elders, community members, and other Knowledge Holders involved in activities related to the Indigenous community’.
European Research Commission. Horizon 2020 “Better Research for Better Health” https://ec.europa. eu/programmes/horizon2020/ sites/horizon2020/files/SPH_ VisionPaper_02062016.pdf	Involving citizens, end- users and the public sector. Citizen Science. Multi- stakeholder approach. Public engagement. Co-design.	‘Science with and for society’ is one of five main priorities in the Horizon 2020 Online Manual for applicants: http://ec.europa.eu/research/ participants/docs/h2020-funding-guide/index_en.htm Science with and for society http://ec.europa.eu/research/participants/ data/ref/h2020/wp/2018-2020/main/h2020-wp1820-swfs_en.pdf	Form requirements vary according to the funding call. Involving patients and citizens is recommended and new approaches are advocated. Citizen science is supported at all stages of research and innovation, with co- design specifically mentioned. Current funding facilitates the establishment of self-sustaining ecosystems for citizen science that will continue beyond the duration of the funds. Guidance encourages institutional changes to sustain new forms of citizen involvement in research and innovation decision making.
UK Health and Social Care Act 2012 endorses public and patient involvement: http://www.legislation.gov. uk/ukpga/2012/7/contents UK Research Councils Concordat for Engaging the Public with Research. UK National Institute of Health Research (NIHR) supports Patient and Public Involvement in research. NIHR Going the Extra Mile strategy. NIHR standards for public involvement are in development.	Public Engagement Public and Patient Involvement (PPI) Co-production.	The Medical Research Council funds public engagement activities https://www.mrc.ac.uk/research/public-engagement/ ‘Pathways to Impact’ covers public engagement activities https://www. mrc.ac.uk/funding/guidance-for-applicants/2-the-application/#2.2.5 Application form guidance has a section on PPI: https://www.nihr.ac.uk/ funding-and-support/documents/current-funding-opportunities/hta/HTA- https://www.nihr.ac.uk/funding-and-support/documents/current-funding-opportunities/hta/HTA-Full-Guidance-Notes.pdf Extensive guidance on budgets and a cost calculator are on the INVOLVE website ( www.invo.org.uk).	All Research Council funding applications have an Impact Summary, which is for the public domain. In addition there is a two page attachment called ‘Pathways to Impact’ where applicants are asked what they will do to make beneficiaries aware of the research and how they will benefit. Impact generating activities are two-way and start when developing the application: https://www.publicengagement.ac.uk/ plan-it/funding/public-engagement-and-pathways-impact. Applicants can request project-specific resources and are asked justify costs. Training costs, specialists in public engagement, materials, venue costs and travel expenses are eligible for full economic costing. There are mandatory PPI sections and a lay summary in NIHR forms. If applicants are a member of the public, patient /service user or carer, they are asked to provide their knowledge, skills and experience instead of a CV. PPI sections ask: Were patients and the public actively involved in identifying the research topic or prioritising the research questions? Were patients and the public actively involved in preparing this application? Please indicate the ways in which patients and the public will be actively involved in the proposed research. Eligible costs include: payment, rewards, expenses, costs of activities and involvement staffing to support, co-ordinate and facilitate involvement. Payment options include fees, vouchers, donations, gifts, funding for training, honorary appointments. Expenses include travel, subsistence, child care/carer costs, personal assistants, overnight accommodation and home office costs. Involvement activity materials, venues, catering and conference fees can be included, as well as translation, interpretation and support for people with impairments. The form requires full economic costs for PPI and justification of costs. There is no upper limit and external peer review guides funding decisions.
US Department of Health and Human Services: Public Involvement with the National Institutes of Health. Patient Centered Outcomes Research Institute (PCORI) standards.	Patient partnerships Patient- Centered Outcomes Patient engagement Co-learning Reciprocal relationships.	Pre-award user guide: https://www.pcori.org/sites/default/files/PCORI- Online-Pre-Award-User-Guide.pdf PCORI Engagement Rubric for the entire research process includes a financial compensation framework https://www.pcori.org/sites/default/ files/Engagement-Rubric.pdf	Researchers partner with patients and other stakeholders from the planning stages through to dissemination of findings to answer patient-centered questions. Focus on outcomes that matter to patients. For some highly technical and methodological projects, patients may not make appropriate partners. A public abstract and an engagement plan covering three stages: planning, conduct and dissemination are required. Applicants are expected to demonstrate how they meet the six PCORI patient engagement principles in their work: reciprocal relationships; co-learning, partnerships, transparency, honesty and trust. Information is required that supports how patient informants and people representative of the population of interest input into decisions about outcomes. Information may come from meetings, surveys or published literature. Applicants are asked to give a detailed budget aligned with engagement activities as outlined in the engagement plan. This includes compensation for patient time and expenses incurred (travel, accommodation, parking, childcare, respite or caregiver expenses. special needs, phone, internet). Applicants are advised not to let cost be a barrier to patient engagement and to include a staff budget to support patient engagement: e.g. recruitment, training, mentoring, co- ordinating and for engagement events.

^*^Websites for international research guidance, definitions of terms and additional information are provided in Box 1, Box 2 and Supplementary File 1

Box 2. **Terminology.**

**Some acronyms for involving people in research**

PPI –Patient and Public Involvement: http://www.ukcrc.org/patients-and-public/. In the European Research Commission, PPI means Public Procurement of Innovative Solutions.

PPIE – Public Patient Involvement and Engagement: https://www.nihr.ac.uk/about-us/documents/PPIE-Leadership/NIHR-PPIE-Strategy_2018-19.pdf

PIA – Public Involvement Activities ^17^

PCORI – USA Patient-Centered Outcomes Research Institute http://www.pcori.org/program/engagement

NIP – National Involvement Partnership which includes the 4PI **–** Principles, Purpose, Presence, Process, Impact which are national involvement standards https://www.nsun.org.uk/FAQs/4pi-national-involvement-standards

**Definitions**

Definitions are derived from the INVOLVE jargon buster ^8^ and international resources in Table 1 and Box 1.

*Participating* in research describes people who have consented to provide data for analysis to further knowledge (participants). Historically participants were referred to as ‘subjects’ of research. ‘Participatory research approaches’ ^18^ is used as an umbrella term which covers ‘participatory action research’ ^19, 20^, co-design ^21, 22^ and co-production of research ^23, 24^. In our opinion, a more suitable umbrella term is ‘partnership approaches’.

*Involving.* INVOLVE ^3^ defines public involvement in research as research being carried out ‘with’ or ‘by’ members of the public rather than ’to’, ‘about’ or ‘for’ them and states that the term ‘public’ includes:

• Patients, potential patients, carers
• People who use health and social care services
• People from organisations that use services

INVOLVE makes a distinction between the ‘public’ and people who have a professional role in health and social care.

The European Union (EU) website refers to ‘citizen Involvement’ which includes upstream priority setting, influencing funding decisions to a more direct downstream involvement of citizens and patients in the use and application of medical knowledge and information. It covers both active citizens who engage from a position of agency as well as those unaware of their contribution ^25^, ‘Citizen Science’ is used as an EU umbrella term which is envisioned as various forms of public engagement with science as a way to promote responsible research and innovation.

*Partnership* is when people who get actively involved in research have a relationship that involves mutual respect and have an equal voice. This contrasts to someone who is consulted occasionally. PCORI consider that the principle is demonstrated when time and contributions of patients and stakeholder partners are valued and demonstrated in fair financial compensation, as well as in reasonable and thoughtful requests for time commitment. When PCORI studies include priority populations, the research team is committed to diversity across all project activities and demonstrates cultural competency, including people with disabilities, when appropriate.

*Reciprocal Relationships* is one of six PCORI engagement principles. They are demonstrated when the roles and decision-making authority of all research partners, including patients, are defined collaboratively and clearly stated.

*Collaborating* is active, on-going involvement in the research process; however, responsibilities are not equally shared like they are in partnerships. Patients may be co-applicants on a grant application, take part in an advisory group or work with researchers to design, undertake and/or disseminate the results of a research project.

*Engaging* is a term used in the USA by PCORI and the Canadian Institutes of Health Research. PCORI define engagement as meaningful involvement of patients, carers, citizens, clinicians and other healthcare stakeholders in the topic selection, design, conduct and dissemination of research findings. There are six PCORI patient engagement principles: reciprocal relationships; co-learning, partnerships, transparency, honesty and trust. The UK National Co-ordinating Centre for Public Engagement in Research ( https://www.publicengagement.ac.uk/do-it) defines public engagement as: ‘the myriad of ways in which the activity and benefits of higher education and research can be shared with the public. Engagement is by definition a two-way process, involving interaction and listening, with the goal of generating mutual benefit’.

*Devolving* is to place decision making in the hands of patients or communities, for example, a community development approach ^26^.

*Consulting* is gaining feedback from patients and communities through e.g. meetings, on-line fora, workshops. The role is considered to be relatively passive when compared to ‘engagement’

*Action Research* brings about improvement or practical change. A group of people who know about a problem work together in a *‘partnership’* to develop an idea about how it might be resolved. They then go and test this idea. The people who take part in the testing provide feedback on their experiences. It has key tenets ^20^:

- Flexible planning – the detailed content and the direction of the research are not determined at the outset
- Iterative cycles with all involved to i) decide what the problem is, ii) decide an action iii) take action iv) learn the lessons from the action v) reconsider the problem and repeat the cycle
- Subjective meanings of those involved determine the content, direction and measures of success of the research
- The research simultaneously improves the situation
- The unique and ever changing social context is taken into account

*Co-production* means people who use services, members of the public and professionals working together in a ‘ *partnership*’ to produce research or service improvement. It is an umbrella term for a concept that means coming together to find a shared solution. ‘Co-‘ can be put before specific research tasks like ‘co-design’, ‘co-build’ and ‘co-construct’. Co-production ^27^ covers the whole research process from idea to dissemination of findings in order to change practice.

*Co-learning* is a term used by PCORI, where the goal is to help patients or other partners to understand research processes. The goal is not to turn patient partners into researchers. PCORI use the term in the context of ‘reciprocal relationships’, where all research partners including patients learn collaboratively. https://www.pcori.org/sites/default/files/Engagement-Rubric.pdf

PPI is put into practice through patients discussing, helping to make decisions and occasionally doing research in order to enhance study relevance, design, conduct and governance. There is no ‘one-size-fits-all’ approach. Flexibility is required to tailor patient involvement to the topic, research question, methods and resources available. This article describes steps that researchers and patient partners can follow when preparing a research funding application ( Box 3). We refer throughout the article to an illustrative example of a researcher who wants to do a study to improve outcomes for patients with migraine, and we provide examples from the literature and authors’ experiences.

Box 3. **Overview of how to involve patients in research.**

A clinician wants to involve patients in a trial of treatment for migraine. Here are steps for involving patients when preparing a research funding application.

1. Understand what patient and public involvement (PPI) is and the different approaches
  i. refer to research funder guidance about public and patient involvement because it varies internationally and is rapidly evolving
  ii. understand how patient involvement differs from patients participating in research
  iii. use language precisely because it varies internationally
2. Find out what research questions are priorities for patients
  i. search the internet for existing work on patient priorities and ask patient organisations
  ii. if patient priorities are unknown, discuss this with your proposed funder and consider how you might fill the gap to progress your research
  iii. prioritise patient-centered outcome measures and find acceptable research methods
3. Identify patients (not your own), charities and/or patient groups to potentially involve as early as possible
  i. consider identifying a professional or lay link worker, perhaps through a charity or a university or hospital patient advisory group
4. Select patients and/or patient groups to be involved in your study
  i. consider equity of opportunity, unheard perspectives and health inequalities
  ii. consider the potential for bias and conflicts of interest
5. Negotiate and agree an approach, tasks and responsibilities at an early stage
  i. consider which approach will add value and rigour to your research
6. Negotiate appropriate funding to pay patients, reimburse expenses, fund activities and staff time to facilitate patient involvement
7. Consider whether training will be required for the proposed roles and responsibilities
8. Consider whether patients or patient groups will ‘do’ any research
  i. do they have appropriate skills?
  ii. how will they add value and are there risks?
  iii. will they be employed?
  iv. who will mentor and provide supervision?
9. Consider the ethical and research governance implications for involving patients in your study
10. Involve patients in writing the grant application
11. Involve patients to plan future reporting and dissemination of your research

## Steps for how to involve patients and the public when applying for research funding

### Understand what patient and public involvement is

At the outset, it is important to understand the theory underpinning PPI. In depth reviews and discussion of theory are available ^1, 4, 10– 13^ and suggest that, depending on the circumstances, PPI will:

- ensure that the research questions and outcomes really matter to patients
- provide perspectives that complement or challenge those of researchers and clinicians
- make research more relevant to the people whom it is designed to benefit
- ensure that proposed research will be acceptable to patients so that they will be willing to participate
- improve the quality of research
- offer lay knowledge that is either independent for the purpose of governance, or specific to the focus of study to enhance its design or conduct
- make research more equitable and ethical, particularly when publicly funded
- improve dissemination to reach wider lay audiences
- increase the likelihood that research will be implemented into everyday practice and impact on patient care
- enable patients to feel that their voice matters.

All of the above could reduce research waste ^14– 16^ if PPI is put into practice in ways that ensure that research is meaningful, acceptable, ethical and useful.

### How does patient involvement differ from patient participation in research?

**Figure 1.**
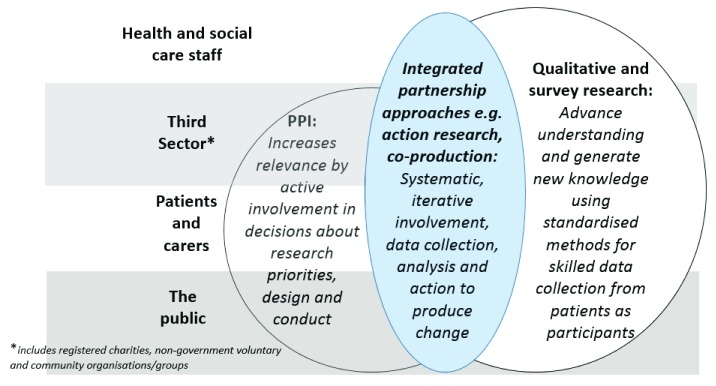
The interface between Public Patient Involvement (PPI), qualitative and survey research across all stakeholders in research.

Patient perspectives can be sought through patient *involvement* and through patients *participating* in surveys, interviews or focus groups to provide data for others to analyse, interpret and act on. The authors have observed that in grant applications and study protocols, PPI is often conflated with qualitative research or patient opinion surveys. Collecting data from patients can be important to gain diverse or representative views, but it is different from PPI and both are often needed ( Table 2). Discussion with patients at a workshop can seem similar to collecting data in a focus group, because both involve listening to patients’ perspectives, but the context and outcomes from listening differ. PPI means that researchers are in a continuing and reciprocal relationship with patients and make decisions with them about the research. In qualitative research, researchers listen to patients in order to improve their understanding of a topic. Focus group discussions or qualitative interviews are audio-recorded and transcribed. Researchers collate, analyse and interpret text data from carefully sampled patients to produce valid new knowledge and generate hypotheses. Qualitative and survey research have systematic methodological quality standards. However, the researcher holds the power and patients may express strong views which may not be reported. In any research, the PPI and the data collection to gain wider patient perspectives can be separate, combined or overlap in some study phases, or they can be completely integrated throughout ( Figure 1). Any combination is possible ( Supplementary File 2, Example 1). They are often combined and integrated in equitable *partnership* research methods like action research, and ‘co-‘ prefixes to research terms, e.g. co-learning and co-production ( Box 2).

**Table 2.** Patient participation and patient involvement in research: Methods compared.

Research methods	Qualitative and survey research:patients are study particpants who provide data (text or numerical) on their perspectives to inform research team decisions.		Public Patient Involvement:patients actively contribute through discussion to decisions about research priorities, design, relevance, conduct and governance from study conception to dissemination	
	Strengths	Limitations	Strengths	Limitations
**Who** **contributes? How** **are they selected** **to represent the** **views of larger** **populations and** **to meet the aims** **and objectives of** **the research?**	Sampling strategies are carefully constructed to recruit patients e.g. for specific characteristics, maximum diversity or a representative sample. Usually participants have little experience of research.	Can be resource intensive. Under-privileged groups can be hard to recruit. When to stop sampling is seldom straight forward.	Individuals provide their own patient experience and perspective. Larger charities have well-developed infrastructure, provide equity of opportunity and empower patients to provide meaningful contributions to multiple projects. Active social media sites access a wide range of perspectives. Patients may undertake training for a PPI role.	Selection processes, characteristics and conflicts of interest are seldom reported. Some PPI groups lobby politicians or have influential networks with key decision-makers. Patients can become professionalised, exclusive and immersed in an academic establishment.
**How many** **contributors are** **required (sample** **size)?**	Variable and flexible in order to answer emergent research questions. Quality standards apply. Small samples may be appropriate if there are qualitative evidence syntheses and few residual uncertainties. Larger representative samples are required where little research exists, to build theory, or if there are multiple uncertainties.	Recruitment problems, lack of resources, limited sites, or convenience sampling can introduce bias. Premature conceptual closure can occur and is more likely when methods tend towards consensus: e.g. focus groups, workshops, Delphi or nominal group techniques.	There is little guidance, so researchers can decide and operate within their available resources. INVOLVE ( http://www.invo.org.uk/) state at least two patient contributors attend team meetings.	Individuals cannot fulfil the role of representing all patients and public perspectives. The resources may limit the number and timing of patient contributions.
**How do they** **contribute to** **each stage:** **e.g. research** **questions,** **design,** **recruitment,** **outcome** **selection, data** **collection,** **process** **evaluation,** **dissemination,** **reporting and** **implementation?**	Focused open research questions e.g. about experience of an intervention/ recruitment. Usually at one time point. Qualitative researchers have highly skilled attention to non-verbal communication, contradictions, language and context. Researchers use the findings to inform decision- making. The patient perspective may or may not inform the research design and conduct. Reporting guidelines apply: http://www.equator-network.org	Researcher skills vary. Closed questions asking “what” rather than “how” or “why” will limit data quality, prime responses and can bias findings. Analysis by inexperienced researchers can generate “common sense” or “so what” critiques rather than skilled interpretation.	Range from limited involvement at steering groups to full engagement as a grant co-applicant satisfying the responsibilities set out by the funder. Methods range from informal discussions to partnership research approaches e.g. action research, co-production. Patient partnerships imply recognition, mutual respect, commitment and equity in decision-making. Patients with appropriate skills may be involved in data collection, analysis or interpretation. There are guidelines for using PPI in trials ^28^ and PPI reporting ^29^.	Protocols and reports often lack transparency for how PPI is operationalised and impacts on decision making. Grant-holder status may be inappropriate for patients with limited resources and can have opportunity costs, e.g. patients working for a charity may have less time for direct patient support. Views can be undervalued. Alternatively views can lead research in a direction that proves counter-productive. Patients may experience regret or blame.
**Ethics, funding** **and research** **governance.**	Data collection and access require ethics committee approval to satisfy the Data Protection Act and Declaration of Helsinki ( http://www.hra.nhs.uk). Participants are not usually paid but can be offered reimbursement for expenses, or a gift to recompense for time or travel. Sponsoring institutions apply research conduct and employment governance to ensure good practice.	Ethics committee approval for study protocols and subsequent amendments are time consuming, and should not be an a priori straight jacket that restricts change. Build flexibility into study protocols for iterative modification of recruitment strategies e.g. if plan A does not work, state plan B and plan C in order to recruit the sample required.	Patient involvement where no data is collected, stored or accessed does not require ethics approval. UK patients are reimbursed for their time ( http://www.invo. org.uk/). There are agreements (of varying formality) about what will be undertaken. Confidentiality is expected. Patients are usually independent of academic institutions and funding bodies. Charities usually have their own ethics and governance policies. Build in flexibility for protocol change when using patient partnership approaches like co-production.	If patients recruit, provide, collect or analyse data, ethics approval is required. If patients do research usually undertaken by researchers, the sponsor’s research and employment governance procedures apply. Short term study funding can result in productive patient partnerships ending. Patient groups often use social media to seek views, which can pose risks for confidentiality and data protection. Patients should consent to how their contribution to research is reported and declare any conflicts of interest.

### Action research, co-design, co-learning and co-production

Action research historically precedes co-production and gathered momentum in the 1940’s as a community-led action in research initiative ^19, 20, 30^. The UK National Institute of Health Research (NIHR) who fund research advocate co-production ^27^ as a method of involving patients meaningfully from start to finish of the research process. Differences in definitions ( Box 2) are subtle, vary internationally and researchers may apply the approaches flexibly in practice. ‘Partnership approaches’ is used in this article as an umbrella term because it acknowledges the changing roles of patients beyond being ‘participants’ or ‘subjects’. Partnership research methods involve patients, clinicians, academics and other relevant stakeholders as equal mutually respected partners in the research team. Being a patient partner implies equal opportunity and equal voice. Equal power in decision-making is sometimes implied, however there are structural and economic power differentials between different types of partner in terms of pay, employment contracts, status and workplace environments. As language is evolving internationally it is more helpful to describe actual patient roles, tasks and responsibilities explicitly rather than use a label for an approach that is open to misinterpretation. For example, co-production ^27^ may mean consulting patients regularly or patients may actively collect and interpret research data. Terms like ‘Participatory Action Research’ confuse because the definition of ‘participation’ in a study means to contribute data, rather than active involvement in research decisions. Partnership research teams decide who has access to participant level data, how to share data securely and how decisions will be made collectively. Partnership approaches can be resource intensive require leadership skills to balance equity of decision-making with a strong scientific rationale. Negotiation skills are required to accommodate different perspectives in order to reach consensus in a timely manner. An important limitation to consider is how the partnership approach is interacting with the intervention: for example action research can become an active intervention component ( Supplementary File 2, Example 2).

*When starting to design a study about migraine, understand how PPI will add value to the research and which uncertainties about patient perspectives might benefit from additional analysis of patient data from a survey or qualitative interviews.*

### Find out what research questions are priorities for patients

Many funders require researchers to justify that their research question addresses what is important to patients ^31– 33^. If a research question is of low priority to the people affected by the condition, or important outcomes are not considered, and/or the intervention in question is considered unacceptable to patients, then further research is wasteful ^11^.

A starting point for researchers is to find out if patients’ priorities already exist for their topic. Many national and international organisations involve patients to identify and publish research priorities specific to a healthcare condition. In the UK, the James Lind Alliance (JLA) specifically identifies and prioritises research questions for funders and there is a register ^34, 35^. JLA establish Priority Setting Partnerships which involve collaborations between patients, carers and clinicians. The NIHR funds JLA advisors and the infrastructure, but a Priority Setting Partnership is responsible for its own funding. The JLA has a guidebook which provides step by step processes to identify research uncertainties and prioritise a top 10 list for different conditions ^36^. Researchers are advised to evaluate how priorities were established and the rigour of processes, as priorities can change with time and some groups may not have had an opportunity to be involved.

If patients’ priorities are unknown, and a Priority Setting Partnership is not available, contacting the potential funder to discuss options may be helpful. When researchers plan bespoke methods to prioritise research, it is important to find patients as soon as possible to identify the topic and refine the research question to ensure relevance. For example: work with a charity or a research organisation to conduct an on-line survey ( Supplementary File 2, Example 3); advertise and run open public workshops with patients to rank research priorities; or ask participants in qualitative interviews what would make a difference, then construct research scenarios for them to ‘think aloud’ which one they would prioritise. Once patients have prioritised the research topic and questions, the next step is to prioritise the outcomes that matter, patient-centered outcome measures and identify acceptable research methods.

*A first step for a researcher is to search the internet for key organisations and guidelines to see if patient research priorities for migraine are available. If not, a researcher can contact migraine charities and talk to a potential funder to seek their advice.*

### Identify patients and/or patient groups to involve as early as possible

Researchers are advised to find people to involve and to plan potential roles, responsibilities and tasks for their study as early as possible. Research teams may approach patients through formal patient groups, charities, community groups, University or Health and Social Care patient advisory panels, national directories such as ‘People in Research’ ^37^, invoDIRECT ^38^, patients who are involved in producing guidelines like The National Institute of Health and Care Excellence ^39^, or through personal recommendation or advertisement. See Supplementary File 2, Example 4. It is usually not considered appropriate to involve patients that members of the research team are currently providing clinical care to ^40^. In the UK, InvoDIRECT ^38^ provides an A-Z on-line resource of organisations, networks and groups that support PPI in health and social care research ( Box 1).

Lay or professional coordinators or link people may help and different sources of patients may be used for different purposes. For example, a head office of a patient charity may be invited to nominate a person to join a study steering committee, whereas a local patient group may help to make recruitment materials appealing and easily understood. Participants in a preparatory survey, focus group or qualitative interview may be invited to volunteer for patient involvement in future research. The qualitative research and PPI then become synergistic.

*A researcher wanting to study migraine could contact a charity, their University or Health Service patient advisory panel or consult directories of patients who are interested in being involved in research. Invite a patient link worker to join the team who will co-ordinate wider patient involvement.*

### Decide who and how many patients to involve

As with any appointment, selection criteria for patients based on the research plan are useful to inform decisions. Deciding the number of patients to involve in a study requires careful consideration. Two is the minimum number recommended by INVOLVE ^3^, however international guidance is less specific. The patient characteristics, skills and numbers will vary according to:

- the study design, e.g. several patients with diverse personal experiences of a health condition may be consulted about which outcomes will be measured in a trial ^41^. Co-authors Arthritis Research UK expect patients to be involved in all applications including lab-based early phase research to develop new treatments
- the prevalence of the condition, e.g. it may be challenging to identify two or more patients with rare conditions
- the relevance and reach of a new intervention, e.g. adverts on Facebook for selected postcodes can identify rural and under-privileged urban perspectives
- how much personal tailoring and choice is possible in the design of the research, e.g. two closely involved patients may advise the research team at meetings for a Cochrane Systematic Review, whereas many diverse patient groups may be consulted when prioritising research questions to improve migraine outcomes.

### Consider equity of opportunity, unheard perspectives and health inequalities

Equity of opportunity for patients to be involved in research underpins UK guidance. The NIHR standards for PPI ^7^ provide practical examples for how researchers can offer inclusive opportunities and sustain respectful, productive relationships. There is a danger that patient contributors are atypical, as the more confident and financially secure are more likely to volunteer. It can be easier to involve older, white and educated people, which can marginalise other perspectives. Health inequalities and equity are important when making research decisions ^42^. Aim to find patients who represent the demographic of those affected by the condition. It can be challenging to access ‘typical’ members of the target population for the specific research question ^42– 44^. See Supplementary File 2, Example 5. An adult or child may be selected to represent their own views ^45^ or, when the research involves children, vulnerable patients or patients with cognitive impairment, then a guardian, relative or carer may represent the patient’s views. A lack of resources can hinder recruiting some patients, such as those from ethnic minorities, the less privileged and less literate. Yet this is important because they tend to experience lower health status and poorer access to services. For these patients it can feel intimidating to meet researchers and attend meetings in a University. Alternative strategies include researchers going out into the community in order to build rapport and trust with patients on their own turf, which can then lead to discussions about research ( Supplementary File 2, Example 1) ^46, 47^. An outreach model for patient involvement via a link coordinator (professional or lay) can help to access less heard perspectives ( Supplementary File 2, Example 5) ^48^. A useful guide for getting started and arranging a meeting with patients is available on the INVOLVE website ^49^.

*A charity partner might help a researcher to plan how patients on low-income or from ethnic minorities can contribute to a research study on migraine. Adverts, social media and attractive visual information in local newspapers and chemist shops may help.*

### Consider the potential for bias and conflicts of interest

PPI in research and political lobbying can co-occur and introduce conflicts of interest with the potential to influence research decisions in ways that have been under-researched ^50^. Researchers are advised to consider sources of funding and affiliations of patient contributors, and to re-assess any arising conflicts of interest during their study.

Patients can work with research teams over many years, attend training courses and become a ‘PPI methodologist’ or expert individual or group. This has advantages and risks. Experienced patients can have an overview of a particular health condition that is invaluable. However, becoming embedded in a research team or an organisation can risk losing the ‘eye of the public’ ^51^. Researchers are advised to consider whether bias due to ‘group think’ ^52^ is possible. This is a risk in any established team, for either researchers or patients to become so familiar with the group or clinical area that they lose sight of fresh perspectives. Selecting new untrained patients for a study can highlight researchers’ preconceptions and assumptions. However, this also has limitations, as it can be difficult for patients to understand, question and challenge researchers when the language and culture are unfamiliar. Patients who have benefited from or experienced adverse events from a particular treatment can introduce bias. Select patients to balance views, for example patients who have positive and negative outcomes from a new procedure or treatment. It may add rigour to include qualitative or survey research to gain diverse and/or representative patient perspectives.

Throughout all stages of a study, researchers and patients make decisions that need to balance and prioritise evidence, personal experiences and competing values held at the individual, family, organisational, political, cultural and environmental levels. Rigour and quality standards for PPI in research are important to counter critics, as there is still some resistance to implementing PPI ^53^.

*A researcher is advised to consider conflicts of interest and sources of bias, for example links to industry or private companies. Seek to balance positive and negative patient experiences relevant to the study.*

### Negotiate and agree an approach, tasks and responsibilities at an early stage

Once patients are involved, it is advisable to agree clear boundaries about the scope of the role, specific tasks and responsibilities. Some flexibility is desirable to accommodate unexpected issues that can arise in research and there are grey areas. See Supplementary File 2, Example 6. The approach can be bespoke for each study or for each phase within a study ^12, 17, 44, 48, 54^ and can vary in the level of patient engagement, responsibility and control. Patients can contribute to three key functions: research decision-making; enhancing understanding of patient experience; and advising how to capture knowledge from other patients. For each function, a question to ask is: which method for involving people will add value and rigour? Example 7 ( Supplementary File 2) draws on the work of Gamble and Colleagues who have produced a useful list of tips for patient roles in clinical trials derived from a cohort study of 111 funded trials ^28^.

Be realistic about what will be possible to achieve and the resources required ^3^. A template for Terms of Reference is available on the INVOLVE website ^49^. Terms of Reference acknowledge the importance of mutual respect, practical communication issues and can be reviewed as the research progresses. Researchers may invite patients to propose ground rules for the length of time required to read and respond to emails and comment on documents, for mutual agreement. It is important for researchers to remember that patients may be managing ongoing health conditions which can be unpredictable. Patients value individual constructive and honest feedback about their contributions in order to learn, gain confidence and maintain motivation ^7^.

*At an early stage a researcher is advised to discuss roles and tasks involved in the migraine study. For example: help to design an appealing patient leaflet, recruit patients, attend project management meetings, interpret findings and present them to lay audiences.*

### Agree appropriate funding for patient involvement

International arrangements for supporting patient involvement in research vary according to the funding opportunity. It is important for researchers to check current guidance for the funding call they are applying to and budgeting guidance is usually available ( Table 1). Negotiate with patients the costs: payment for patient time, any special needs (e.g. childcare, hearing impairment, translation services), training, reimbursement of travel and subsistence expenses. In addition, include costs for staff time to co-ordinate, support, train and facilitate patient involvement. Researchers are advised to spell out to patients the best case and worst case scenarios (e.g. delays to study start and finish), and what contributing to the study would and could involve. Some patients prefer to volunteer, others prefer cash payment or vouchers. Consider patients who are less financially secure. Patients may rely on benefits, part time work or retirement pensions, therefore consider how difficult it is to pay upfront for travel, to scan travel tickets in order to claim research expenses or to have access to computers or printers to access documents for a meeting.

Preparatory PPI activity prior to submitting the grant application can pose a problem for researchers because funding for this is seldom available prior to a grant. Yet this is precisely when patients can have important impact on the study research question, design and plan. In England, the NIHR Research Design Service will provide small amounts of money to cover PPI at the design stage ^55^. Some Universities fund generic patient partnership panels (e.g. 56) to work with researchers who are seeking funding and larger charities can often help ^57^.

*When costing a study about migraine, negotiate sufficient funds to pay for the planned PPI activities, be realistic about the workload and the resources required and consider special needs.*

### Training for patients involved in research

Providing or offering training may or may not be appropriate depending on the patient role and the purpose of training. Training may be desirable in order to undertake highly skilled roles like reviewing grant applications or sitting on independent trial steering committees. In particular, training in the principles of evidence based medicine, with consideration of where and how patient stories fit in evidence hierarchies may be useful. Example 8 (see Supplementary File 2) provides some training programmes that support patient involvement in research. For patients new to a PPI role, support to develop their abilities and confidence to express their views and question researchers may be relevant. Many universities, research funders and charities provide learning and support activities.

There are many PPI tasks where training is not necessary, where a different perspective is what really matters and patient experience of a healthcare condition is the required expertise. For example, when helping to choose important outcomes or advising on patient information or recruitment strategies, ‘untrained’ patients may make particularly valuable contributions.

### Patients doing research

Traditionally, academics with qualifications, experience and recognised research skills collect and analyse data. However, increasingly patients are helping to recruit participants, collect or analyse data and some UK grant application forms ask about this ( Supplementary File 1, Section B). Such questions arguably prime researchers to think that all boxes should be ticked, without considering the implications. Only appropriately trained patients or lay people should undertake research. Shared experiences of a condition can build trust, empathy and a bond which may help to recruit difficult to engage groups, for example children in care ^45^. However, attention is required to individual expertise, training requirements, supervision and the scientific rigour necessary to execute high quality research. Patients may do research alongside researchers in partnership research methods ^58^ and a paradigm of patient-led research is emerging facilitated by social media and digital technologies ^59^. INVOLVE has a Patient-Led-Research-Hub to support patients who want to pursue their own research ideas ^38^.

In the UK, any researcher accessing study participants who are NHS patients or staff requires a letter of access, sometimes referred to as a ‘research passport’, obtained from the NHS Research and Development offices ( Supplementary File 2, Example 9) ^60^. If patients or lay people help to recruit participants to research, gain informed consent or collect, share or analyse data from individual or group discussions, qualitative research or surveys, then they are ‘doing research' and there are potential governance implications for the sponsor of the research in terms of employment law, ethics, leave entitlement and indemnity. Researchers should not encourage patients to do research because it requires less resource, or because it obviates the need for relatively costly skilled researchers whilst simultaneously bypassing regulatory hurdles. Rather, researchers and patient partners can decide together whether patient researchers are appropriate and beneficial to specific research projects.

*Researchers wanting to study migraine may consider the pros and cons of patients doing aspects of the research and the governance issues.*

### Working together ethically

Consider how to work with patients ethically. PPI can be empowering for individuals and communities, but there are tensions and risks, including exploitation ^25^, and the burden and resource implications can be considerable ^10^. Some ethical principles for researchers to consider when involving patients in research include:

- avoiding discrimination, undue persuasion, excessive burden or creating a sense of obligation to be involved in the study
- the distribution of power in research
- valuing patient contributions and fair financial compensation
- conflicts of interest, research integrity and respect for intellectual property
- the confidentiality of data and protecting anonymity of research participants
- advancing science through honest and accurate reporting.

INVOLVE ^3^ states that UK ethics committee approval is not required when patients advise research teams, prioritise research questions, make choices relating to design, share decision-making or disseminate research findings. However, there can be grey areas particularly in relation to defining ‘data collection’. NHS or University Ethics committee approval is required in the UK if personal information, i.e. data as defined in the Data Protection Act ^61^, is collected, shared and stored for future analysis and reporting. For iterative partnership research approaches like co-production, the current ethics committee processes create many challenges ^62^. Researchers can request informed consent from participants to share anonymised data with patient partners, so that they can be involved in analysis and interpretation as members of the study team.

There are international differences in requirements for research ethical and governance approvals, and particular challenges with digital health research ^25^ which are beyond the remit of this article. New EU General Data Protection Regulation ^63^ commenced in May 2018, and requires transparency about the source of personal data, the purpose and who data will be shared with.

Audio-recording of PPI meetings in order to write accurate but not verbatim minutes, does not require ethics committee approval. However, it does require at least verbal consent from all present at the start of the meeting and the recording should be destroyed as soon as the minutes are agreed. People should receive forewarning of the intention to audio-record, know the purpose, what will happen to the recording and to the content, and be able to object or withdraw. If audio-recordings are stored for longer than is necessary, transcribed verbatim or if there is an intention to report or publish potentially identifiable quotations or content arising from PPI activities, then ethics committee approval is required. Ethics committees have lay committee members, who consider the ethical issues relating to patient involvement.

*A researcher wanting to study migraine should consider the ethical issues when involving patients in the design and conduct of their study. Consider patient burden, equity and power, fair and respectful arrangements, confidentiality and the purpose, processes and consequences of any data collected or stored.*

### Involve patients in writing a grant application

Patients sit on research prioritisation committees and funding panels, alongside clinicians and academics, to decide which research is commissioned and which grants are awarded. See Supplementary File 2, Example 10. Many UK funding panels expect to read convincing and meaningful accounts of how patients have had an impact at key stages: preparatory work to inform the planned research; writing the application form particularly the lay summary; and the proposed PPI activity during the study. Expect to be challenged if PPI appears tokenistic. It is important to consider the trade-offs between specifying a plan for PPI in a research protocol and building in some flexibility for change as the research progresses. This may be challenging in countries where regulatory approvals for amending protocols is time consuming.

Patients can help researchers to write the whole grant application in an engaging, easy to understand language. The lay summary is often one of the first sections in a grant application that funding committee members read to gain an overview of the study. Reviewers like to understand exactly what study participants will experience from start to finish. Describe PPI clearly so that the reader understands who, why, how many, how often, what methods and what impact patients have already had on the grant application and will have in contributing to future research decisions. For example, decisions about recruitment methods, intervention delivery or components, which outcomes will be primary or secondary and how to collect data. It helps to use language precisely and to understand how *involving, participating, collaborating, consulting and engaging* with patients in research differ ( Box 2).

*A patient helping to write and edit a grant application can make it clear what will happen to patients who participate and how patients will be involved from study conception to dissemination of findings.*

### Plan future dissemination of findings

Patients can advise on how research might have an impact on health and health care beyond an academic audience. They often have in-depth knowledge of their condition and of on-line sources of information beyond that of academics and clinicians. They can help to write reports, blogs or summaries of findings creatively. See Supplementary File 2, Example 11. Offering participants a lay summary of the research findings is good practice. ‘Patients Included Charters’ provide accreditation for involving patients in conferences and in journal publications ^64^ and GRIPP2 PPI reporting guidelines ^29, 65^ are available. Involvement of patients and the public is a critical component in successful implementation of research findings into healthcare, although evidence for best practice is limited ^3, 66, 67^.

*The grant application for a study about migraine may propose a public event with a charity to present the results of the study. Researchers and patient partners may give joint talks. Small group discussions with migraine patients can suggest ways to spread the news and change care.*

## Conclusion

This article provides a starting point for researchers and patient partners who are planning to seek funding for research. There is no current international consensus on best practice or terminology and guidance is evolving across countries and research disciplines. A crucial distinction when gaining patient perspectives is between patient *involvement* in research and patients *participating* by providing data in surveys, qualitative interviews or group discussions. The ethical governance implications differ particularly regarding data protection.

Researchers and patient partners can choose a wide range of different approaches to PPI and each study will require consideration of the optimal approach. Rigour is needed because patients’ lived experience and persuasive narratives can influence important research decisions and the outcomes are not always predictable. Evidence is needed about how different methods of involving people can improve research decisions, healthcare outcomes and impact. A more collaborative and reciprocal partnership approach with patients has the potential to ensure that research undertaken matters to a wider tranche of society and involves those who stand most to benefit from it.

## Key messages

Important questions for researchers about including PPI in their research:

• How can I find people in society (patients, patient groups, carers, the taxpaying public, lay organisations) who can make important contributions to research design, conduct and dissemination?
• How will PPI help me to access the perspectives of those who the research potentially will impact on?
• How can different approaches to involving patients as consultants, collaborators or partners improve the relevance, quality, future implementation and sustainability of research?
• How can patients contribute to three key functions: research decision-making; enhance researchers’ understanding of different perspectives; and knowledge capture?
• How can PPI, qualitative research and surveys of patient opinion be optimally combined?

## Data availability

No data are associated with this article.

## Author information

PH wrote the first draft. All authors have contributed to and approved the final version. PH and IB are members of the NIHR/HTA Commissioning Board and General Board respectively and AOC was a member of the NIHR Programme Grants for Applied Research panel 2007–2017. JLD is an emerita NIHR Senior Investigator and was previously on the NIHR HTA Commissioning Board, NIHR HSR Board and CRUK Population Health Board. JT has lived with rheumatoid arthritis for over 30 years and is also a carer for a brother with schizophrenia. She has been involved in PPI for 7 years covering the whole spectrum from basic science to applied health services research. Her background is in higher education and she works part time for the Open University. CM is the research manager for Arthritis Research UK, a registered charity in England and Wales no. 207711, Scotland no. SC041156. PH and AP have worked as a General Practitioner and as a Physiotherapist respectively. AP is an associate editor with Cochrane Stroke and has received funding from Cochrane Training to synthesise evidence relating to PPI in systematic reviews ^68^. PH has been a chair and deputy chair of a research ethics committee. PH is guarantor and affirms that the manuscript is an honest, accurate, and transparent account of the analysis reported.

## Supplementary material

Supplementary File 1: Section A, Additional terminology and international information relevant for patient and public involvement in research at the funding application stage; Section B, Questions asked by UK National Institute for Health Research (NIHR) on funding application forms.

Supplementary File 2: Examples of different approaches to incorporating patient and public perspectives into research design and conduct.
