# Supplementary material for: How to incorporate patient and public perspectives into the design and conduct of research
Source: F1000Res. 2018 Jun 18;7:752. [Version 1] doi: 10.12688/f1000research.15162.1 (PMC6192439; doi:10.12688/f1000research.15162.1)
Supplement: Supplementary file 1 [file f1000research-7-16517-s0000.tgz › f99aac73-19bb-4375-8ab5-d25f515ad35f.docx]

**Supplementary File 1**

**Section A. Additional terminology and international information relevant for patient and public involvement in research at the funding application stage.**

**Stakeholder involvement**

Camden and colleagues^1^ define stakeholders as potential ‘knowledge users’ such as patients and people whose primary job is not directly in research. This includes:

- Patients, their families and caregivers
- Individuals representing patient groups
- Policy makers
- Clinicians

Concannon and colleagues^2^ identify that it can be challenging to ensure that all relevant groups of stakeholders are involved in research, and developed the ‘7Ps framework to identify stakeholders’. This framework defines the following seven groups of stakeholders whom researchers should consider involving in their research:

1. Patients and the public
2. Providers (i.e. health professionals providing care)
3. Purchasers (including employers, insurers, government organisations)
4. Payers (e.g. the public; insurers)
5. Policy makers
6. Product makers
7. Principle investigators (other researchers and their funders)

**Knowledge user engagement**

Canadian Institutes of Health Research use the term ‘knowledge user’, which refers to, but is not limited to, a practitioner, policy-maker, educator, decision-maker, health care administrator, community leader, or an individual in a health charity, patient group, private sector organisation, or media outlet.^3^  Citizens, primarily affected populations and community organisations, are all identified as knowledge users.

**Consumer involvement**

The Cochrane Consumer Network includes the following definition of consumer: *‘A healthcare consumer is any actual or potential recipient of health care’*.^4^ Healthcare consumers who use Cochrane evidence are generally patients, carers and family members, or people interested in remaining healthy, who are seeking information about a health condition or treatment for personal use. Cochrane’s Consumer Network, also includes members of patient advocacy groups and others with an interest in the organisation of healthcare, such as practitioners seeking patient involvement in health research. Consumer involvement is a term also used by The Australian Government National Health and Medical Research Council (NHMRC).^5^

There are difficulties in relation to the use and definition of the term consumer. It may not have universal acceptance or understanding. The term ‘consumer’ may not be considered appropriate among some groups of people or in some countries. It is associated with buying or choosing a health service. Other terms may be preferred, such as user or receiver of health care, patient, a member of the public, citizen, carer/caregiver, or lay person. Each of these terms have different connotations in different environments. In addition, the definition of consumer is sometimes expanded to include all ‘users’ of research, including health professionals, policy makers and other organisations.^4^

**Patient orientated research**

Patient orientated research is the phrase used by the Canadian Institutes of Health Research (CIHR) to refer to a continuum of research that engages patients as partners, focusses on patient-identified priorities and improves patient outcomes. This research, conducted by multidisciplinary teams in partnership with relevant stakeholders, aims to apply the knowledge generated to improve healthcare systems and practices. The Canadian Strategy for Patient Orientated Research (SPOR)^6^ has a Patient Engagement Framework^7^ which has guiding principles for patient engagement:

- ***Inclusiveness:*** Patient engagement in research integrates a diversity of patient perspectives and research is reflective of their contribution – i.e., patients are bringing their lives into research.
- ***Support***: Adequate support and flexibility are provided to patient ‘participants’ to ensure that they can contribute fully to discussions and decisions. This implies creating safe environments that promote honest interactions, cultural competence, training, and education. Support also implies financial compensation for their involvement. (Note, the term ‘participants’ as used in the Patient Engagement Framework is used in a different context to the definition in Box 2 used in our article)
- ***Mutual Respect***: Researchers, practitioners and patients acknowledge and value each other’s expertise and experiential knowledge.
- ***Co-Build:*** Patients, researchers and practitioners work together from the beginning to identify problems and gaps, set priorities for research and work together to produce and implement solutions.

**Canadian Institutes of Health Research** **distinguish between patient engagement and citizen engagement**

*Patient engagement* in research occurs when patients meaningfully and actively collaborate in the governance, priority setting, and conduct of research, as well as in summarizing, distributing, sharing, and applying its resulting knowledge.^8^

*Citizen engagement* includes interested representatives from the general public, consumers of health services, patients, caregivers, advocates, and representatives from affected community and voluntary health organizations. Citizens are "engaged" when they play an active role in defining issues, considering solutions, and identifying resources or priorities for action. This "meaningful involvement" can take place at a variety of stages in the research, planning, or implementation phases of a project.^9^

**Including more vulnerable and marginalized patients, who are sometimes referred to as ‘hard to reach’**

‘Hard to reach’ is an expression used by researchers for people who are harder to find, recruit and involve and who have perspectives that are less often voiced. A peer reviewer of a previous version of this article recommends the following references which consider involvement more broadly than partnership, and provide helpful suggestions:

- Bonevski B, Randell M, Paul C, *et al*. (2014)^10^
- Dugas M, Trottier M-È, Chipenda Dansokho S, *et al*. (2017)^11^

**Some other useful references:**

A peer reviewer of a previous version of this article suggested these additional references:

- Bagley HJ, Short H, Harman NL, *et al*. (2016)^12^
- Crocker JC, Boylan AM, Bostock J, *et al*. (2017)^13^
- Pandya-Wood R, Barron DS, Elliott J. (2017)^14^

**Section B. Questions asked by UK National Institute for Health Research (NIHR) on funding application forms in 2017 at the time of writing this article (see** [**http://www.nets.nihr.ac.uk/programmes/hta**](http://www.nets.nihr.ac.uk/programmes/hta)**).**

**Please note, application forms are being revised**

**Please indicate the ways in which patients and the public will be actively involved in the proposed research.**

- Design of the research
- Management of the research (eg steering / advisory group)
- Developing participant information resources
- Undertaking / analysing the research (eg member of research team)
- Contributing to the reporting of the research
- Dissemination of research findings
- Other

**References**

1. Camden C, Shikako-Thomas K, Nguyen T, *et al*. Engaging stakeholders in rehabilitation research: a scoping review of strategies used in partnerships and evaluation of impacts. Disability and Rehabilitation 2015;**37**(15):1390-400.

2. Concannon TW, Meissner P, Grunbaum JA, *et al*. A New Taxonomy for Stakeholder Engagement in Patient-Centered Outcomes Research. Journal of General Internal Medicine 2012;**27**(8):985-91.

3. CIHR. Knowledge user engagement. <http://www.cihr-irsc.gc.ca/e/49505.html>. Last accessed 7th December 2017

4. Cochrane Consumer Network. Cochrane Consumer Network. <http://consumers.cochrane.org/>. Last accessed 7th December 2017

5. National Health and Medical Research Council. Consumer and community involvement. 2017. <https://www.nhmrc.gov.au/research/consumer-and-community-involvement>. Last accessed 7th December 2017

6. CIHR. Canadian Institutes of Health Research (CIHR): Strategy for patient-oriented research (SPOR) - patient engagement Ottawa, Canada. 2014.

7. CIHR. Strategy for patient-oriented research - patient engagement framework: Canadian Institutes of Health Research,<http://www.cihr-irsc.gc.ca/e/48413.html> (accessed 7th December 2017). 2014.

8. CIHR. Patient engagement. 2014. <http://www.cihr-irsc.gc.ca/e/45851.html>. Last accessed 7th December 2017

9. CIHR. Citizen engagement. 2012. <http://www.cihr-irsc.gc.ca/e/41592.html>. Last accessed 7th December 2017

10. Bonevski B, Randell M, Paul C, *et al*. Reaching the hard-to-reach: a systematic review of strategies for improving health and medical research with socially disadvantaged groups. BMC medical research methodology 2014;**14**:42.

11. Dugas M, Trottier M-È, Chipenda Dansokho S, *et al*. Involving members of vulnerable populations in the development of patient decision aids: a mixed methods sequential explanatory study. BMC Medical Informatics and Decision Making 2017;**17**:12.

12. Bagley HJ, Short H, Harman NL, *et al*. A patient and public involvement (PPI) toolkit for meaningful and flexible involvement in clinical trials – a work in progress. Research Involvement and Engagement 2016;**2**(1):15.

13. Crocker JC, Boylan AM, Bostock J, *et al*. Is it worth it? Patient and public views on the impact of their involvement in health research and its assessment: a UK‐based qualitative interview study. Health Expectations : An International Journal of Public Participation in Health Care and Health Policy 2017;**20**(3):519-28.

14. Pandya-Wood R, Barron DS, Elliott J. A framework for public involvement at the design stage of NHS health and social care research: time to develop ethically conscious standards. Research Involvement and Engagement 2017;**3**(1):6.
