# Supplementary material for: How to incorporate patient and public perspectives into the design and conduct of research
Source: F1000Res. 2018 Jun 18;7:752. [Version 1] doi: 10.12688/f1000research.15162.1 (PMC6192439; doi:10.12688/f1000research.15162.1)
Supplement: Supplementary file 2 [file f1000research-7-16517-s0001.tgz › 542fd4a7-3542-4f85-867d-dcf1f5711c86.docx]

**Supplementary File 2.**

**Example 1. Combining PPI, qualitative and survey research to inform decisions about controversial publicly funded interventions.**

**Pat Hoddinott**

**Background:** Financial incentives to motivate behaviour change to improve future health are controversial. Some consider incentives as bribery, paternalistic and morally indefensible and there may be opportunity costs, for example funding cuts for other health services. Understanding the diversity of public perspectives is therefore important.

**Aim:** To combine partnership patient involvement with recent mothers who smoke and don’t breastfeed with mixed methods research. The overall aim was to build a research platform to inform the design of incentive interventions to improve smoking cessation during pregnancy and breastfeeding outcomes.^1^

**Methods:** A Health Visitor, her manager and a Children’s Centre Manager identified two mother and baby groups in under-privileged areas, and acted as co-ordinators between the groups and the research team. They negotiated group involvement, roles, responsibilities and funding and were the named contacts on the funding application. Ethics Committee approval was sought from the start in recognition of the power differentials and the grey area between providing data and collaborating on the grant. As trust developed, the group communicated directly via text and Facebook to arrange monthly meetings with nominated researchers who led the PPI.

PPI contributions from the mother and baby groups were combined with qualitative research in order to include a wide range of experiences about smoking in pregnancy, infant feeding and life circumstances.^2^ Theoretical, purposive and maximum diversity sampling strategies were applied according to the salient research questions at each study stage.^1^ The PPI groups helped researchers to recruit women with views that are less often voiced. This is a similar approach to the ‘Outreach Model’ described in the RAPPORT study (see Example 5).^3^

The mother and baby groups also contributed to the design and conduct of systematic reviews and to a UK wide surveys of the general public, women who have smoked and health professionals.

**Results:** PPI groups provided perspectives that were different from those heard from research participants who participated in qualitative interviews.^2^ Some women in the PPI groups provided informed consent to formal qualitative interviews, so that disconfirming data was included in the qualitative analysis. The PPI groups contributed to several changes to the study design and conduct which are reported elsewhere.^2^ In particular, women told us that they could not afford to buy a breast pump and that a free pump might act as an incentive for and women like them to try breastfeeding. As a result, the definition of ‘incentive’ was broadened and systematic review inclusion criteria included trials that provided a free breast pump. A free breast pump was the most acceptable incentive for breastfeeding amongst the UK public and health professionals.

**Discussion:** PPI perspectives were combined and integrated throughout the study with qualitative interview data collection, surveys of the UK public and health professionals, systematic reviews and mixed methods data analysis.

**Reflections:** Mutual trust between researchers and women in groups whose voices are less often heard was established over a period of time. The researcher going to the mother and baby groups rather than expecting them to come to the University facilitated trust and engagement. The mother and baby groups contributed missing voices from the target population and perspectives that were honest and not heard in the qualitative interviews. Combining PPI with mixed method data collection to gain wider perspectives benefited the rigour and quality of the research.

**Example 2. A cautionary tale for partnership approaches to patient and health professional involvement in research design: using action research to design an intervention to improve breastfeeding rates**

**Pat Hoddinott**

**Background:** There is a problem as recent trials in the UK and Australia have reported no significant difference in breastfeeding rates for interventions providing additional breastfeeding support, despite extensive Cochrane systematic review evidence that additional support is effective.^4^ Intervention design decisions are therefore not straight forward.

**Aim:** To design a complex intervention to improve breastfeeding outcomes, to pilot the intervention and conduct a definitive effectiveness trial

**Methods:** A breastfeeding coaching pilot study was designed using an action research approach which involved monthly audio-recorded meetings with front line midwives, health visitors, women, a health promotion specialist and a service manager.^5,6^ A health visitor who facilitated a breastfeeding support group identified and recruited two women with children who joined the team. A local member of a childbirth charity also joined the team. Health professionals were invited and recruited to the research team who were involved in facilitating local support groups for mothers and babies. There was uncertainty about whether one-to-one peer coaching or group based peer coaching would be the most promising intervention to take forward to a full trial. Both interventions were evaluated in a before and after study, which surveys of acceptability and qualitative interviews with women participating and not participating in the intervention. Health professional facilitated group based peer support was more acceptable and showed promise for evaluation in a full trial.

**Results:** The team members valued the action research process and the opportunity to be involved in a new local initiative to support breastfeeding. The full trial of the intervention did not show a significant difference for a new policy to provide breastfeeding support groups compared to existing services.^7^ The trial was embedded within qualitative research to ensure that a wide range of participating and non-participating participants and professionals could share experiences of the trial. Data analysis revealed that the successful component of the pilot study was likely to be the action research approach.^8,9^

**Discussion:** Care therefore needs to be taken when undertaking intervention development, feasibility and pilot studies to avoid action research methods, or other partnership approaches like co-production and co-design, becoming an active intervention component.

**Reflections and impact on subsequent research:** For the design of the next trial, qualitative researchers conducted interviews with a diverse sample of women living in disadvantaged areas (who are less likely to breastfeed) every month from pregnancy until 6 months after birth. The research team drew on the longitudinal qualitative data analysis and the research evidence to construct seven intervention vignettes which were typed onto cards and shown to women at the final interviews.^10^ Women were asked to think aloud, compare and discuss their views and the interviews were audio-recorded, transcribed and analysed. Some partners and significant others were involved. The intervention describing a dedicated feeding team was unanimously the most popular. This resulted in the promising FEeding Support Team (FEST) pilot trial.^11,12^ The FEST study steering group had patients involved. The FEST intervention has fixed and flexible components, so that some aspects of intervention delivery could be tailored to local context. Care was taken to avoid action research methods becoming part of the FEST intervention.

**Example 3. How patients prioritised pain as a topic, which resulted in a commissioned call for arthritis research**

**Jane Taylor**

An example from my experience would be Arthritis Research UK's 'Pain Challenge' (<http://www.arthritisresearchuk.org/research/information-for-applicants/types-of-grant/pain-challenge-2018.aspx>). Arthritis Research UK commissioned a large survey of 2500 patients with arthritis. The results of this survey demonstrated that pain was not being addressed enough in research. So the charity committed themselves to prioritise funding of research into pain and set up two major workshops in London to try and identify patients’ research priorities (<http://www.arthritisresearchuk.org/research/information-for-applicants/types-of-grant/pain-challenge-workshop.aspx>). This has led to the creation of a Pain Roadmap.

Patients were an integral part of these workshops and indeed facilitated roundtable discussions with other patients, researchers, healthcare professionals and other funders. The insight gained from those workshops shaped the 2017/18 pain call (<http://www.arthritisresearchuk.org/research/information-for-applicants/types-of-grant/pain-challenge-2018.aspx>) in terms of research emphasis. There is a smaller pain working group which also has patient representation to ensure good continuation of patient involvement here.

This is very much a work in progress, adapting year by year and patients are involved in ongoing discussions. Patients sit on the funding committees that the research bids come to so they can act as a check on the agreed priorities. This is an interesting example because the initial impetus came from patients as subjects of a large study and their voices on what matters to them changed the direction of research within the charity. Patients then were involved in a different capacity as equal partners in helping to set research goals / priorities.

**Example 4. A patient perspective on a European-wide study**

**Jane Taylor**

APPROACH is a five year ongoing study with 24 European centres and one from the USA (see [www.approachproject.eu/approach](http://www.approachproject.eu/approach) ). The main purpose is to create a central database, which combines biomedical data from 10,000 patients with osteoarthritis and healthy people. This would allow for stratification of patients into subtypes of disease which will enable more personalised treatment through improved diagnostic tools and drug development.

There is a Patient Council with 8 members from different countries who advised on the initial bid and are involved in advising on two of the four work streams. For example, we have been involved in developing the protocol for the clinical study, devising patient information leaflets and consent forms. We attend the annual European conference, give a presentation at this event and contribute to the newsletters.

However one of the issues here is that it has been difficult to recruit members of this Patient Council from countries in Southern Europe such as Spain because the culture of patient involvement is not as developed there as it is in the UK, Germany and Holland for example. This is important because the study involves a two-year longitudinal study of patients from four countries, to validate the data. Two of these countries, Spain and France are not represented on the Patient Council. The patients were initially found through patient organisations such as Arthritis Research UK <http://www.arthritisresearchuk.org/> and Reumafonds in the Netherlands <https://home.reumafonds.nl/> so these countries are represented as well as Norway and Germany. There are language barriers also: as the language of the whole project conferences is English, it is easier for patients like myself whose first language is English or who are relatively fluent in English to participate.

There are some cultural differences and expectations even between the different members of the current Patient Council, yet despite this patients are vocal and are managing to work together and maintain ongoing dialogue through a patient online platform and regular teleconferences with the research team. There is an annual conference where the patients work on practical issues with the relevant scientific group. This face to face contact is really important. The project has emphasised for me again the value of the process of patients and scientists working together through relevant aspects of the project rather than patients simply commenting on a finished product. Designing a patient protocol together in a room allows for the kind of dialogue that changes thinking and expectations on both sides and leads to a better end result.

**Example 5: Outreach model for involving patients in research**

Wilson and colleagues^3^ in the RAPPORT study provide case studies for different approaches to involving patients in funded studies for six conditions: diabetes, cystic fibrosis, arthritis, dementia, public health and learning disabilities. They describe an outreach model, where a patient may be involved in project management meetings and then go out into the community to engage with patient groups in the target population.

Examples describe a trial with outreach to a group of parents and children who have cystic fibrosis and systematic reviews involving parents of children with severe cognitive disabilities. Activities include parents showing leaflets to children and asking for suggestions on how to improve recruitment. The children suggested changes to the design and words like ‘mortality’ and ‘lung function’. Outcomes from the PPI include: improved recruitment, outcomes that mattered to parents, more sensitive wording in questionnaires; parents setting the inclusion criteria for a systematic review and a lay presentation at a conference.

The authors emphasise the importance of sufficient time to build social collaborative relationships and the importance of fun activities. A limitation with the outreach mode is its potential to be tokenistic. A ‘middle person’ can operate to endorse a study with the target population, when in fact the study ideas and decisions have been made by the research team.

**Example 6: Involving patients and the public in a systematic review**

**Alex Pollock**

**Background:** PPI within systematic reviews has been proposed as a way to make reviews more useful and enhance the uptake of evidence into practice.^13^ Cochrane, an international organisation which produces systematic reviews of healthcare evidence, has had patient and public (described by Cochrane as ‘consumer’) involvement as an explicit principle of the organisation since it began in 1993.^14,15^ However, despite this, there are considerable variations in PPI within systematic reviews,^16^ and a lack of information about the best ways of involving people in reviews.^17^

An update to a Cochrane Systematic Review of physiotherapy for people with stroke^18^ was planned. However problems had been experienced during the previous version of the review in relation to the categorisation of different approaches to physiotherapy, and it was necessary to address these problems and clarify the focus and scope of this systematic review.

**Aim:** to engage key stakeholders in an update of a Cochrane systematic review of physiotherapy treatment approaches for patients with stroke,^18^ in order to ensure clinical relevance of the completed review.

**Methods**: A “stakeholder group”, comprising 13 purposively selected stroke survivors/carers, physiotherapists and educators, was formed. Three pre-planned stakeholder group meetings were held, each with a clearly identified aim. At each meeting, nominal group techniques were used to reach consensus on a number of pre-agreed topics or statements. This involved a period of discussion around a statement or issue, followed by voting on paper slips to indicate level of agreement/disagreement. The proportion of stakeholders agreeing with each statement was determined, and consensus reached on a decisions relating to the structure and presentation of the review. Each of the meetings was audio-recorded, transcribed verbatim and securely stored. Data were used to support related publications (e.g. ^19^). Ethical approval was granted, and stakeholders gave consent for use of data collected at the meetings.

**Results:** At the first meeting, stakeholder group members reached consensus (using nominal group technique) on two statements; 11/13 members considered that the categorisation in the previous version of the Cochrane review was not appropriate or clinically relevant, and 100% of members agreed that evidence relating to Chinese physiotherapy approaches should be incorporated into this systematic review. At the second meeting, stakeholder group members agreed a new taxonomy to describe physical rehabilitation interventions within the systematic review. At the third meeting, stakeholder group members agreed the key implications for practice arising from the evidence synthesised within the review. The wording of these implications was agreed and was included within the published review.

**Discussion:** The stakeholder group was involved in the update of this Cochrane review, using clearly described structured methods to reach consensus decisions. This involvement gave the stakeholders considerable control over the review focus and methods, and impacted substantially on the presentation of the review results. Methods and results of this involvement have been fully reported elsewhere. ^19^

**Reflections:** This example illustrates how attempts at PPI resulted in a blurring of the boundaries between PPI and qualitative research. This involvement was planned, and subsequently presented within publications, as ‘PPI’. ^19^ However the use of formal group consensus methods based on nominal group techniques, arguably strayed into the territory of ‘research’. The method resulted in collection of both qualitative and quantitative data which were later presented within reports and research papers. The decision to seek consent from the stakeholders for use of the audio-recorded data for research purposes arguably placed the stakeholders in a position of being research participants, rather than PPI contributors. Nevertheless, the aims and methods adopted handed the stakeholders considerable control over aspects of the systematic review; a role which is not aligned to *participants* of research, and suggests *involvement*.

**Example 7: Continuous and responsive patient involvement in clinical trials^20^**

Gamble and Colleagues^21^ describe the following approaches used in trials:

*Continuous involvement in management and/or oversight of research*. This could include patients as research co-applicants on a research grant, and/or members of study steering committees and/or PPI advisory groups. There is variation in how management or oversight roles are operationalised. For example, some Universities have dedicated PPI advisory groups which the research team can go to with questions, or who provide oversight. Patients who are not members of University advisory groups may also attend the project management group and spontaneously raise new issues or challenge views expressed by the researchers.

*Responsive involvement for specific tasks*. Patients may be responsible for writing a lay summary of the study or a patient information leaflet. Alternatively researchers may produce a first draft which is then edited by patients into lay language. Involvement may be one-to-one, a group discussion, a stakeholder workshop or on-line.

Continuous and responsive involvement are commonly combined, where an advisory group provides oversight throughout the research process, in addition to more active responsive PPI at a specific stage of a study or for a specific task. Patients involved in the oversight and responsive roles may be the same or differ. For example Pollock 2015^19^ had a PPI group who contributed to three meetings within the research process and also had an oversight role throughout, whereas Coon 2016^22^ had an advisory group involved throughout the project, and an additional four PPI events at key stages in the research.

**Example 8: Training programmes that support patient involvement in research**

The European Patients’ Academy (EUPATI) provides on-line ‘Mini-course starter kits’ for patients (and people who train patients) who want to develop skills to become involved in medicines research. The website includes materials in a variety of formats including presentations, videos and quizzes. For example, a patient who is working with a research team who are applying for funding, could gain skills in designing a research protocol <https://www.eupati.eu/clinical-development-and-trials/mini-course-starter-kit-protocol-design/>

INVOLVE ([www.invo.org.uk](http://www.invo.org.uk)) provides case studies that aim to help researchers and patients share knowledge, skills and experiences. There is an events page and examples of how to run your own in house training and support, run courses, provide mentorship and facilitate peer learning.

The NIHR ‘I am Research’ website (<https://www.nihr.ac.uk/news-and-events/support-our-campaigns/i-am-research>) provides free on-line training courses, an events page and links to social media to help patients understand scientific research.

Arthritis Research UK run training workshops on patient involvement and communication throughout the year and across the UK. Training days are co-facilitated by patients who share their experiences of involvement in research. For more information on upcoming sessions or to sign up, please contact [patientinsight@arthritisresearchuk.org](mailto:patientinsight@arthritisresearchuk.org)

The Cochrane training ‘involving people’ resource helps researchers to involve people in systematic reviews: <http://training.cochrane.org/involving-people>. It has useful on-line sections on ‘Finding people to involve’ with examples and case studies.

**Example 9: How research governance impacts on PPI helping to set the research agenda for preterm birth**

**Sandy Oliver**

There can be some conflation of purpose and methods when it comes to actually engaging with people. A large PPI exercise was undertaken to set a research agenda around preterm birth that included professional organisations and patient organisations. Because the families at greatest risk of preterm birth tend to be socio-economically disadvantaged, they tend to be less active in patient organisations’ activities. Such families were therefore specifically sought out, just to ask their ideas about what research they’d like to see, or what unanswered questions they had. Because the most likely place to find women from these families was on NHS premises, NHS ethics approval was required. This reduced the time available to seek their input. Consequently, exactly the people whose views would be most important were the people whose participation was limited by the research governance requirements.

**Example 10. A patient perspective on improving involvement in the research review process**

**Jane Taylor**

In 2015 Arthritis Research UK called for the research community to submit bids on the alleviation of pain and fatigue. This was in response to a large commissioned survey of patients on what most affected their quality of life. They realised that pain and fatigue were key issues and yet the research focus was on cure of the disease rather than managing pain and fatigue.

Patient involvement in research was already well established within the charity with a ‘user’ committee consisting of patients, carers and healthcare practitioners, who met three times a year to review research bids and advise the scientific committees on what they felt should be funded. The charity used this research call as an opportunity to test out a different way of working and to ensure that patients who were already part of their involvement group were made much more central to this review process.

A single ‘Quality of Life’ panel was formed comprising equal numbers of scientific experts and patients. Everyone had access to each full research bid as well as the lay summary, and lay reviews were also carried out by patients from the pain centre at Nottingham University. The panel was co-chaired by myself (JT) as a lay (patient) chair as well as a scientific chair and we both worked together taking it in turns to chair the meeting. Seating was organised so scientists and patients were mixed up and patients always spoke first about a particular bid to summarise the research, reviews, applicants’ rebuttal and give their overall opinion. This was then followed by a scientific lead for that bid and then a general discussion. Patient reviewers and academic reviewers, some of whom were initially sceptical, perceived this way of working as successful. There was parity in numbers, discussion and everyone had an equal say in the outcome. What was interesting about this initial call was that many of the bids were scientific rather than applied health services research and yet it still seemed to work. The charity has now adopted a variation of this model for use in all its research calls including basic science.

**Example 11. The Men’s Health Forum charity wrote a booklet to inform services about how to help men to lose weight**

**Pat Hoddinott**

The lead researcher, Professor Alison Avenell at the University of Aberdeen invited the Men’s Health Forum charity to be co-applicants in a series of systematic reviews about the evidence for weight management in men with obesity.^23^ Four charity representatives from England, Scotland and Ireland attended three workshop days with the research team during the study and commented on review documents by email in between meetings. They were co-authors on the final report to the funder. At the end of the study, a member of the Men’s Health Forum Charity led a collaboration with Public Health England and translated the review findings into a practical and visually attractive booklet entitled ‘How to make weight loss services work for men’^24^ which is being widely used in community settings.

**References**

1. Morgan H, Hoddinott P, Thomson G, *et al*. Benefits of Incentives for Breastfeeding and Smoking cessation in pregnancy (BIBS): a mixed-methods study to inform trial design. Health Technology Assessment 2015;**19**(30):1-522.
2. Morgan H, Thomson G, Crossland N, *et al*. Combining PPI with qualitative research to engage ‘harder-to-reach’ populations: service user groups as co-applicants on a platform study for a trial. Research Involvement and Engagement 2016;2(1):7.
3. Wilson P, Mathie E, Keenan J, *et al*. ReseArch with Patient and Public invOlvement: a RealisT evaluation – the RAPPORT study. Health Services and Delivery Research Southampton, UK: NIHR Journals Library 2015.
4. Renfrew MJ, McCormick FM, Wade A, *et al*. Support for healthy breastfeeding mothers with healthy term babies. The Cochrane database of systematic reviews 2012;5:CD001141-CD41.
5. Hoddinott P, Lee AJ, Pill R. Effectiveness of a breastfeeding peer coaching intervention in rural Scotland. Birth 2006;33(1):27-36.
6. Hoddinott P, Chalmers M, Pill R. One-to-one or group-based peer support for breastfeeding? Women's perceptions of a breastfeeding peer coaching intervention. Birth 2006;33(2):139-46.
7. Hoddinott P, Britten J, Prescott GJ, *et al*. Effectiveness of policy to provide breastfeeding groups (BIG) for pregnant and breastfeeding mothers in primary care: cluster randomised controlled trial. BMJ 2009;338.
8. Hoddinott P, Pill R, Chalmers M. Health professionals, implementation and outcomes: reflections on a complex intervention to improve breastfeeding rates in primary care. Family Practice 2007;24(1):84-91.
9. Hoddinott P, Britten J, Pill R. Why do interventions work in some places and not others: A breastfeeding support group trial. Social Science & Medicine 2010;70(5):769-78.
10. Hoddinott P, Craig L, Britten J, *et al*. A prospective study exploring the early infant feeding experiences of parents and their significant others during the first 6 months of life: what would make a difference? : NHS Health Scotland,http://www.healthscotland.com/documents/4720.aspx. 2010.
11. Hoddinott P, Craig L, Maclennan G, *et al*. The FEeding Support Team (FEST) randomised, controlled feasibility trial of proactive and reactive telephone support for breastfeeding women living in disadvantaged areas. BMJ Open 2012;2(2).
12. Hoddinott P, Craig L, MacLennan G, *et al*. Process evaluation for the FEeding Support Team (FEST) randomised controlled feasibility trial of proactive and reactive telephone support for breastfeeding women living in disadvantaged areas. BMJ Open 2012;2(2).
13. Wallace J, Nwosu B, Clarke M. Barriers to the uptake of evidence from systematic reviews and meta-analyses: a systematic review of decision makers’ perceptions. BMJ Open 2012;2(5).
14. Bastian H. The power of sharing knowledge: Consumer participation in the Cochrane Collaboration UK Consumer Centre: Cochrane Collaboration. 1994.
15. Gartlehner G, Flamm M. Is The Cochrane Collaboration prepared for the era of patient-centred outcomes research? [editorial]. Cochrane Database of Systematic Reviews 2013;3.
16. Boote J, Baird W, Sutton A. Public involvement in the systematic review process in health and social care: A narrative review of case examples. Health Policy;102(2):105-16.
17. Domecq JP, Prutsky G, Elraiyah T, *et al*. Patient engagement in research: a systematic review. BMC Health Services Research 2014;14(1):89.
18. Pollock A, Baer G, Campbell P, *et al*. Physical rehabilitation approaches for the recovery of function and mobility following stroke. Cochrane Database of Systematic Reviews 2014;4.
19. Pollock A, Campbell P, Baer G, *et al*. User involvement in a Cochrane systematic review: using structured methods to enhance the clinical relevance, usefulness and usability of a systematic review update. Syst Rev 2015;4:55.
20. Gamble C, Dudley L, Allam A, *et al*. Chapter 8, Discussion and conclusions. An evidence base to optimise methods for involving patient and public contributors in clinical trials: a mixed-methods study. Southampton, UK: NIHR Journals Library, 2015.
21. Gamble C, Dudley L, Allam A, *et al*. Patient and public involvement in the early stages of clinical trial development: a systematic cohort investigation. BMJ Open 2014;4(7):e005234.
22. Coon JT, Gwernan-Jones R, Moore D, *et al*. End-user involvement in a systematic review of quantitative and qualitative research of non-pharmacological interventions for attention deficit hyperactivity disorder delivered in school settings: reflections on the impacts and challenges. Health Expect 2016;19(5):1084-97.
23. Robertson C, Archibald D, Avenell A, *et al*. Systematic reviews of and integrated report on the quantitative, qualitative and economic evidence base for the management of obesity in men. Health technology assessment (Winchester, England) 2014;18(35):v-vi, xxiii-xxix, 1-424.
24. Men's Health Forum. How to make weight-loss services work for men. Somerset, England: Haynes Publishing Group.
