# Supplementary material for: How to incorporate patient and public perspectives into the design and conduct of research
Source: F1000Res. 2018 Jun 18;7:752. [Version 1] doi: 10.12688/f1000research.15162.1 (PMC6192439; doi:10.12688/f1000research.15162.1)
Supplement: Peer review history

# Peer review: Referee response for version 1

**DOI:** 10.5256/f1000research.16517.r39232

**Published:** 2018-11-12

**Authors:** Clare Jinks

**Affiliations:** 1 Research Institute for Primary Care & Health Sciences, Keele University, Keele, UK

Thank you for the opportunity to review this paper. The aim of this paper is to discuss how researchers can involve patients when applying for research funding, and to outline opportunities and pitfalls. It uses a combination of knowledge about this process from literature and the authors' wide ranging experience.

 This article provides a general background to patient and public involvement in research. The article very usefully includes in Box 1 and Table 1 current resources and guidelines that are available. The information is very comprehensive and relevant to the aims of the article - which is how to to incorporate PPI into the design and conduct of research. This overview is unique and in my view will be widely used. However can it be moved to later in the article? The tables do break up the flow of the paper and break up what I think is an important section about the difference between PPI and patient participation in research. 

 The section on the difference between patient involvement and patient participation in research is much needed as PPI is often conflated with qualitative research. This is still a common problem. I wondered if Figure 1 could come earlier, or if Box 2 can show the differences more explicitly instead of providing a list of definitions, can the differences be displayed side by side, using maybe column headings of differences and similarities. This will enable the reader to access the important distinctions more easily.

 On page 10 in the section about action research, co-design and co-learning there is a sentence about how a partnership approach may interact with an intervention. I think this is the first time intervention studies are mentioned as previous text has stated that patient participation in research may be through interviews, focus groups and surveys. I wondered if this could be expanded to include that these can be undertaken in the context of trials, as then the text would align better with the material in the supplementary documents. 

 There are a lot of examples in the supplementary files and this can be quite difficult to navigate. 

 I have an observation on how the steps are characterised. At first I thought the text in italics are the recommended steps for researchers? For example on page 11 the first step for researchers outlined is to search the internet for organisations and guidelines to see if research priorities have been established in their area of interest. There is however some advice given earlier (written in italics) that recommends understanding what PPI will add and which uncertainties about patient perspectives may benefit from more exploration.

 Then I realised that the steps are characterised in the blue subheadings. These are a mix of 1. advice  (e.g. understand what PPI is (this is a conceptual issue)), 2. questions (e.g. how does PPI differ from patient participation?) and then 3. labels for different issues (e.g. patients doing research, which is a practical issue). Could there be more consistency across the paper? I wondered if it would work if they were all translated into specific statements / recommendations particularly as the aim is to provide guidance on how to incorporate PPI.

 Related to the above - I wondered whether the section headings of "how does PPI differ from research participation" and action research, co-design, co-learning and co-production" should be at the same level as the others. They are very important - but I think are fundamentally about understanding what PPI is. 

 The text in italics is also a mix I think of statements of principles - e.g. seek to balance positive and negative views, rather than a statement of how to do that (the title of the paper is how to incorporate PPI....) 

 The section on how many patients to involve is again really helpful as this is the question that people often ask. This is usually asked in the context of "representativeness" of the patients' views. Can this section be expanded - with some examples of what teams can say when the "representativeness" of their patient advisors is questioned. It links to a later section on bias and conflict of interest. 

 The authors recommend that researchers select patients with balanced views. It is difficult to know what view people hold at the outset. Can this recommendation again be expanded upon - with ideas about how you may do this, or how you may get fresh perspectives over time.

 Towards the end there is a heading about involving patients in writing a grant application. This made me realise that the advice spans both the process of PPI at pre-award and then outlining what to do post award. I wondered therefore whether this section should come earlier. PPI advisors will have an input into the design of the PPI throughout and the research design throughout, and therefore impact on decision making about earlier topics in the article. 

 I think this is a really valuable paper in providing such a comprehensive overview of resources and guidelines for PPI and in highlighting the key challenges of good PPI practice. My suggestions are really about structuring the content to make it more accessible to the reader by highlighting maybe two aspects. First the need to understand what PPI is and is not. Second the practical issues - how to do it.

I have read this submission. I believe that I have an appropriate level of expertise to confirm that it is of an acceptable scientific standard.

# Peer review: Referee response for version 1

**DOI:** 10.5256/f1000research.16517.r39231

**Published:** 2018-11-02

**Authors:** Kathryn M Sibley, Patricia Roche

**Affiliations:** 1 George and Fay Yee Centre for Healthcare Innovation, Winnipeg, MB, Canada; 2 George and Fay Yee Centre for Healthcare Innovation, Winnipeg, MB, Canada

Thank you for the opportunity to review this manuscript. This article gives an overview of steps research teams and patient partners can take when working together to prepare a research funding application. We believe a guide for inclusive and meaningful involvement of patients and public partners, particularly in the planning stages of research, is a highly useful and timely resource for health researchers. We thought it was appropriate that the authors included a variety of resources from different continents, and appreciated the attempt to summarize the varied (and often confusing) terms and concepts that exist around patient and public involvement. It was great to see involvement of patient partners in authorship of the paper. We also wonder if patient partners have been involved in providing review comments as well. 

 We did struggle with the overall flow of the article, due to its non traditional structure, thereby making it challenging to follow for the reader. Despite the valiant attempt to describe differences in terminology around patient and public involvement in research and key concepts that need to be considered for meaningful patient and public involvement, terms are at times dispersed, seem to be used interchangeably throughout the article, and do not follow a step-by-step order that would be expected from an overview of ‘steps’ in a process. In addition, many key considerations that should be made prior to engaging patients or members of the public are left to the end of the article (such as training for patients involved in research, or working together ethically). In general, the article operationalizes PPI into a series of steps and although mentioned at some points later in the article, the theory and principles of equity and social justice that are critical to meaningful and inclusive engagement are presented later in the article. However, to ensure researchers are incorporating these principles, we suggest they be introduced sooner and have a more central role as the lens through which PPI is viewed. We feel it would be more beneficial to begin with terminology and key concepts, and then provide an overview of the subsequent steps involved in engagement.

 Given that the article appears to be an attempt to synthesize information and provide an overview of the steps to take when involving patients and the public in preparing a research grant application, a clear description of the methods used to gather, sort, and summarize the sources and information contained in the article is missing. Such information is critical for demonstrating the strength and rigor of the recommendations. Although we recognize this is an opinion article that is not intended as a formal synthesis, we feel that including a description of the literature review methods would provide a landmark for others who may seek to undertake a full synthesis in the future. 

 The tables and boxes included are excellent resources for researchers seeking to learn more about PPI and how it is done in different contexts and regions. However, aside from the definitions, their placement may be better suited to the supplementary material, rather than embedded within the article itself (especially Table 1, as it is in landscape orientation making it difficult to read in digital form). 

 As we have heard from our stakeholders and patient and public partners, language is a critical aspect of ensuring that PPI is successful, inclusive, and respectful. Many of the decisions that should be made through dialogue with patient partners are presented as decisions for the research team to make and share with patient partners. We recommend directing researchers to dialogue with patient and public partners about topics such as ground rules, preferences for compensation, preferences for feedback, and other matters in which patient partners are involved, in order to ensure that decision-making power in engagement activities is equally shared. A specific example is on page 12 under the heading “Agree appropriate funding for patient involvement”, where the authors state that researchers “Negotiate with patients the costs: payment for patient time, any special needs…” and “Researchers are advised to spell out to patients the best case and worst case scenarios…” –  we caution to consider carefully how language can challenge or perpetuate power differentials that often tend to place the researcher above the patient as having more expertise and education. We suggest that terms like ‘discuss’ and ‘dialogue’, in place of ‘negotiate’ and ‘spell out’, can help work towards equality and mutual respect in the research partnership. Some specific points the authors may want to address are detailed below. 

**Page 3:**

 Given that the concept of PPI is central to the article, a definition (or a discussion of the variability between definitions) should be one of the first points of discussion. 

 The authors state that “PPI includes patients, potential patients, families, carers, patient groups and members of the public who use or have access to health and social care services”. We would suggest PPI also includes those who may be currently unable to use or access health and social care services.

 The authors discuss ‘patients’ including “people who do not describe themselves in this way”, an important consideration for ensuring inclusive and meaningful engagement – however, we feel the discussion of why people may not describe themselves as ‘patients’ (for example, medicalization of people with disabilities, stigma attached to living with a mental health issue).

**Box 1:**

 It would be of great interest to us to know how the resources listed in this box were chosen and why. We also believe that given the iterative nature of many online resources and the frequency with which URLs change, it may be best to avoid including hyperlinks for specific documents, many of which will likely become defunct within a year’s time. 

**Box 2:**

 Under ‘some acronyms for involving people in research’, it is unclear why this particular handful of terms was chosen, yet we do not see common acronyms such as PE (patient engagement; https://bcsupportunit.ca/patient-engagement-methods-cluster) and PAR (participatory action research ^1^) . 

 The section of definitions appears to lack structure or hierarchy and is confusing in its presentation. Rather than clarify terms, this box seems to further complicate the differences in language used. Specifically, ‘partnership’ in engagement should also include participatory action research and community-based participatory research ^2^; devolving would also seem to fall under ‘partnership’; ‘consulting’ is described as “relatively passive when compared to ‘engagement’”, but still falls within the realm of ‘engagement’. 

**Box 3:**

 This overview is an excellent concept and will be highly useful to research teams looking to engage with patients in their work. However, we feel that there may be some information not found here that may be useful. This includes a step for assessing capacity of both research teams and potential patient and public partners to meaningfully engage – for example, determining the willingness of the research team to change directions based on patient and public partner input; determining what resources are available to support engagement activities; and determining the underlying purpose for engaging.

 Point 3 indicates researchers should identify patients to involve “as early as possible” – this may be unclear to those unfamiliar with the PPI process, and should be more explicit – i.e. engaging before the research questions and methods have been determined – something we do not see mentioned elsewhere in this article.

 Under 3.i, it is suggested that researchers consider identifying a “professional or lay link worker”, however many research teams will not (for whatever reason) choose to hire an outside professional to assist with engagement activities, and will embark on it themselves. For this reason, we feel it is critical to mention the need for reflexive practice and trauma-informed approaches for those who are not familiar with these approaches and their need when engaging with people with lived experience of health issues, and the potential retraumatization associated with sharing those experiences. 

 Under 5, there is a minor grammar error – “agree an approach” should read “agree *on *an approach”. 

 Under 6, we believe the use of language such as ‘negotiate’ may perpetuate power imbalance between research teams and patient and public partners. This language posits researchers and patients on opposing sides, when perhaps what the authors intended to suggest with ‘negotiate’ was a method of working together to determine appropriate funding and compensation – with which we agree. Perhaps using a term such as ‘researchers and patient partners should work together’ instead of ‘negotiate’ would be more appropriate. In relation to this point, it seems that a key component missing from this article’s discussion of compensation for patient and public partners is determining the preference of patients and *actually asking them*what they want in terms of level of involvement, supports, payment, etc. Similarly, under 8, the authors suggest that researchers “consider whether patients or patient groups will ‘do’ any research” and subsequently assess their skills, ability to add value, status as employees, and mentorship/supervision – we again suggest that these considerations should be made *with *patient and public partners, rather than decided for them. 

**Page 10:**

 From the title of the section “Action research, co-design, co-learning and co-production”, it seemed as though the authors would be providing a discussion of these different principles, but instead was a discussion of the differences between definitions that should probably have been included at the beginning of the article. We would suggest moving this information to before the ‘steps’ of involvement are discussed, and potentially changing the heading since it is somewhat misleading given the content of the section that follows. Additionally, the first sentence discusses the historical context of ‘action research’, yet this is the only mention of the chronology of terms and seems somewhat out of place. Without discussion of the origin of other terms mentioned in the article, we would suggest that this is unnecessary to the reader’s understanding of concepts. 

 Under “Action research, co-design, co-learning and co-production”, there is a minor grammatical error – in the sentence “Equal power in decision-making is sometimes implied, however there are structural and economic power differentials between different types of partner in terms of pay…”, the word “partner” should be pluralized to read “partners”. 

 The authors have brought up a critical consideration regarding power differentials between patients, however the discussion of these and other systemic power imbalances seems to be somewhat lacking in the article, particularly regarding the dynamics of relationship-building in research partnerships, where researchers may hold more ‘power’ and need to engage in reflexive practice to understand how this impacts the process of engagement. We also recommend a discussion of safe spaces, a key consideration for addressing power imbalances in research partnerships. As a reference, we would suggest reviewing the 2017 BMC Health Services Research article by Shimmin et al. ^3^.

 In the second column on this page, the sentence “Partnership approaches can be resource intensive require leadership skills…” appears to contain a grammatical error and should perhaps read “Partnership approaches can be resource intensive and require leadership skills” or “Partnership approaches can be resource intensive, requiring leadership skills”. 

 The authors mention “negotiation skills are required to accommodate different perspectives in order to reach consensus in a timely manner”, however we are not sure that this aligns with the principles of meaningful and inclusive PPI in research – though consensus and timeliness are obviously of importance to researchers, we are not convinced that these are central tenets of engagement, and would argue that respect for patients’ stories and lived experiences should be valued over ‘timeliness’. 

 Under “Find out what research questions are priorities for patients”, the authors state that “If a research question is of low priority to the people affected by the condition, or important outcomes are not considered, and/or the intervention in question is considered unacceptable to patients, then further research is wasteful”. Although we do not disagree, this is a bold statement that we would suggest tempering, given that basic, fundamental research is often of low priority to patients, and may not have a direct intervention for treating disease or improving health, though it often serves as critical foundations for future discoveries. 

**Figure 1:**

 The figure demonstrates the people involved in different types of activities. It seem as though the integrated partnership approaches are intended to be positioned as the ‘overlap’ between PPI and qualitative and survey research. Generally, we found this figure is somewhat difficult to interpret and aesthetically unpleasing. We also question the exclusion of health and social care staff from PPI, and wonder why people in these roles would not be able to participate in PPI. 

**Page 11:**

 In determining patient priorities, we are pleased to see mention of JLA, an important methodology. We also think this would be an excellent opportunity to introduce the Patient-Led Research Hub, mentioned later in the article.

 The use of survey methodology as an example of identifying patient priorities seems overused – there is a lack of discussion of other potential priority-setting methods (such as patient journey mapping and digital storytelling). This is particularly important for researchers who are new to the concept of PPI who would benefit greatly from information about alternatives to the survey methodology. 

 Mention of hard-to-reach groups should be mentioned initially under “Identify patients and/or patient groups to involve as early as possible”, to ensure it is at the forefront of researchers’ minds when considering their recruitment strategy. 

**Page 12:**

 Under “Consider the potential for bias and conflicts of interest”, we would recommend mentioning that this should be a consideration for discussing ground rules and/or terms of reference. This would also be a good time to mention that the research team and patient partners should discuss together what to do in cases of potential conflicts of interest, and how those will be addressed in the context of the research partnership. 

 Within the heading “Negotiate and agree an approach” there appears to be a word missing, such that the heading should read “Negotiate and agree *on *an approach”. Additionally, in the first sentence of the paragraph under this heading, “Once patients are involved, it is advisable to agree clear boundaries…” should read “Once patients are involved, it is advisable to agree *upon *clear boundaries…”. 

 In discussing the ‘scope of the role’, it is also important to note that the role of the researcher in the partnership should also be discussed, and this should be indicated in the article. 

 The authors state that “Patients can contribute to three key functions” – we would argue that patients often contribute to other functions such as collecting data, interpreting results, and informing knowledge translation activities, to name a few. 

 The authors state that “Patients value individual constructive and honest feedback about their contributions in order to learn, gain confidence and maintain motivation.” Although this statement may be true, the indicated reference is a link to a NIHR site declaring the development of national standards for PPI (from March 2017). This statement in particular should be supported by a reference, or replaced with the suggestion that research teams discuss with patient and public partners how they want to receive feedback and what they need to learn, gain confidence, and maintain motivation. 

 The heading “Agree appropriate funding for patient involvement” seems to be missing a word, and should probably read “Agree *on *appropriate funding for patient involvement.”

 The sentence “Negotiate with patients the costs: payment for patient time” should also include expertise, a major contribution of patients to the research process, such that this part of the sentence should read “payment for patient time and expertise.”

**Page 13:**

 The inclusion of consideration for patients not having to pay upfront for travel and having access to computers and printers is critical and we commend the authors for including it. 

 In discussing the steps for PPI in grant development, discussing how to budget for these early activities would be appropriate and useful for those reading this article. At the very least, a reference to an existing budget tool (such as INVOLVE’s cost calculator - http://www.invo.org.uk/resource-centre/payment-and-recognition-for-public-involvement/involvement-cost-calculator/) would be appropriate and highly useful.

 Under “Working together ethically”, it would be helpful to include references for researchers to explore these concepts in more detail – how to ensure distribution of power in research, perhaps guides to reflexive practice questions (such as Shimmin et al.’s article), guidelines for valuing patient contributions and fair compensation (such as those produce by the SPOR National Disease Networks).

**Page 14:**

 The authors mention “Audio-recording… does not require ethics committee approval” – we are unsure whether this advice is accurate for all potential jurisdictions, and would suggest including a note about inquiring with the readers’ own regulatory bodies would be appropriate here. 

 The authors warn “Expect to be challenged if PPI appears tokenistic”, but provides no guidance for what this means and how to avoid it. We would suggest including more detail and/or a reference.

 The authors state that “It helps to use language precisely and to understand how *involving, participating, collaborating, consulting and engaging *with patients in research differ” - this statement gives the impression that these concepts are mutually exclusive, when in fact they are overlapping and intertwined. Perhaps this could be restated as “how *involving, participating, collaborating, consulting and engaging *with patients in research differ, and what the overlap between these concepts is.”

 In the Conclusion, the authors call for evidence about the engagement process, and we would suggest they may also want to touch on the potential to have patient and public partners included as participants (in evaluating the engagement process), and should discuss this when issues around ethics are discussed (page 13) and/or when comparing partners and participants (page 3).

We have read this submission. We believe that we have an appropriate level of expertise to confirm that it is of an acceptable scientific standard, however we have significant reservations, as outlined above.

## References

[1] BaumFMacDougallCSmithD: Participatory action research. *J Epidemiol Community Health*.2006;60(10) : 10.1136/jech.2004.028662 854-7 10.1136/jech.2004.028662 16973531PMC2566051

[2] HolkupPATripp-ReimerTSaloisEMWeinertC: Community-based participatory research: an approach to intervention research with a Native American community. *ANS Adv Nurs Sci*.27(3) :162-75 1545557910.1097/00012272-200407000-00002PMC2774214

[3] ShimminCWittmeierKDMLavoieJGWicklundEDSibleyKM: Moving towards a more inclusive patient and public involvement in health research paradigm: the incorporation of a trauma-informed intersectional analysis. *BMC Health Serv Res*.2017;17(1) : 10.1186/s12913-017-2463-1 539 10.1186/s12913-017-2463-1 28784138PMC5547533

# Peer review: Referee response for version 1

**DOI:** 10.5256/f1000research.16517.r39334

**Published:** 2018-10-16

**Authors:** Sally Crowe

**Affiliations:** 1 Crowe Associates Ltd, Oxford, UK; 2 Cochrane, London, UK

Thank you for the opportunity to review this article – which I think is an important mile stone publication in the business of patient and public involvement in the activity that prepares and contributes to research applications.  This is a timely and useful review of the literature and the experiences of the research authors, one day we might look back and realise that investing in this stage of research yields benefits and savings in research down the line.

 I was interested in the reasons for the authors writing this article and was not surprised to see that it was about the conflation of qualitative research and patient and public involvement in research.  This article helpfully, and in a practical way helps to ‘de couple’ the most pertinent issues in this domain.

 The use of an illustrative example in migraine research helps to bring the review of evidence and discussion in each section to a useful and practical conclusion for the reader, and could be easily re interpreted for their own research context.

 It is also a strength in this article that research is considered as both primary studies and research synthesis and reviews, both of which require careful patient and public involvement

*Page 3* – in the section *How does patient involvement differ from patient participation in research?*

 “the context and outcomes from listening (to patients) differ. PPI means that researchers are in a continuing and reciprocal relationship with patients and make decisions with them about the research. In qualitative research, researchers listen to patients in order to improve their understanding of a topic”.  This is probably the most helpful sentence I have read in a while!  The further discussions about the choices of researchers that may or may not include qualitative perspectives in their analysis further underlines that in PPI the power dynamic is different and it is an important difference. 

*Figure 1 is helpful*

 I have a problem with the word iterative – and would suggest a plainer language option especially as the rest of the language used in the diagram is of the non-research variety.

*Table 2*

 I struggled with this table maybe because I am not a researcher – I would prefer to see the differences between the two rather than the strengths and limitations of each but can appreciate that for researchers making choices this might be very helpful information and analysis.

*Boxes 1 and 2*

 For an article such as this I think that the *contents of Box 2* are more helpful for readers untangling what is meant by PPI in pre funded research and *would put Box 1 as a supplementary file* – this information is more easily found for a curious researcher and Box 2 really adds value to the article as a whole as authors have collected the (sadly) rather large collection of terminology used in PPI and sought to differentiate it.  I like the fact that the authors have stated and used their preferred terminology and articulated the reasons for their choice (partnership approaches).

*Table 1*

 I imagine this could be immensely useful to researchers and it is a useful comparison tool but positioning in the middle of the narrative is a shame I think as it breaks up the flow of reading.

*Find out what research questions are priorities for patients*

 I think that there is a step before initiating priority setting exercises that encompass PPI – increasingly there are published accounts of priority setting that may or may not include the perspectives of patients and the public.  There is also an emerging checklist to support the quality assessment of these accounts and specifically the degree of PPI in them

- Tong A., Sautenet B., Chapman JR., Appraisal checklist used in a systematic review of priority setting partnerships in health research (Currently being reworked as a priority setting reporting checklist and will be renamed REPRISE Checklist). 

 

*Consider equity of opportunity, unheard perspectives and health inequalities*

 This is a particularly important and pertinent section and offers practical advice and ideas for researchers, especially the use of outreach models that offer more scope for addressing these issues more directly, but may put researchers out of their comfort zones.

*Consider the potential for bias and conflicts of interest*

 This feels an underwritten first paragraph.  It feels more important to instigate transparency and declaration of financial and other interests *for all* research participants – than the more in-depth description of the ‘experienced patient?’.  Additionally some text about how to manage these conflicts of interests in patients/public may really help readers address this issue.

*Agree appropriate funding for patient involvement*

 There are important considerations here especially around equity of involvement – its good to see these spelled out.

*Training for patients involved in research*

 I was very pleased to see this in the text for this section “For patients new to a PPI role, support to develop their abilities and confidence to express their views and question researchers may be relevant.” A much under-appreciated aspect of PPI, I would suggest that it doesn’t just apply to those new to a role who may find it harder (as am embedded part of the research team) to challenge the research orthodoxy…

 Also the training needs to be two way – for research teams as well as involved patients and the public.

*Key messages Important questions*

 I like these but I would include a challenging first question to researchers – ‘Why do they want to do PPI?’

 I think that there is an important aspect of self-discovery in PPI in research and it helps to understand self/organisational motivations to doing this preparatory work.  These reasons may encompass rational or outcome-based reasons (which the current list addresses) but it is also important to understand motivations on a human and relational level.

I have read this submission. I believe that I have an appropriate level of expertise to confirm that it is of an acceptable scientific standard.

# Peer review: Referee response for version 1

**DOI:** 10.5256/f1000research.16517.r35143

**Published:** 2018-07-02

**Authors:** Kristin Liabo

**Affiliations:** 1 National Institute for Health Research (NIHR) Collaboration for Leadership in Applied Health Research and Care South West Peninsula (PenCLAHRC), Medical School, University of Exeter, Exeter, UK

This article gives a comprehensive overview of the involvement of patients, carers and members of the public in health research. It is well written and addresses some important thorny issues in regards to involvement. The article is likely to be useful for researchers new to involvement. Also, as someone who has been facilitating involvement for some time it is very useful to see all this information pulled together. Partly due to the comprehensiveness, I suggest some of the information in boxes and supplements could be cut down. The sheer volume of information might confuse novice readers.

 For example, the authors point to the many different and overlapping terms used in this field. For a new author it might be more helpful if the information about all these terms was reduced so that the article focuses on the substantial differences rather than the terms used. I found boxes 1 and 2 quite overwhelming to read through at the beginning of the article. Perhaps it would help if they were supplementary files instead of in-text?

 Another example is the definition of ‘participating’ – I found the introduction to participatory action research here quite confusing because this can be a study design where involvement and participation happen in tandem or are intersected.

 Overall, I would have liked to see less of the detailed information on various websites and terms, and more incorporation of the thorny issues, e.g. where research and involvement intersect, and discussion about what we can do about this. It is these aspect of the article that are most interesting, in my view. But the attention to this would depend on the purpose of the article.

 Some other comments:

 In Box 3, which gives an overview of the involvement process, I would suggest not using the term bias, because this is commonly used for samples. I agree with the point made – and it is important as often ignored in involvement guidelines – but I think it would be better not to use bias and instead say something about whether the topic of the research is contentious amongst different groups of patients (or something to that effect).

 Also in Box 3, bullet number 7 mentions roles and responsibilities. I would suggest the agreement of these needs to come much earlier in the process. For example, this could come under point 1 – when the researcher familiarises themselves with involvement. What roles would they like patients, carers or members of the public to have?

 I really like Tables 1 and 2 – these are super useful overviews. I am not clear what purpose Supplement 1 has, this relates to my previous point on the brevity and detail of information.

 In Supplement 2 I find some of the examples lacking in purpose and clarity beyond saying that involvement can happen. I don’t find that most of the examples provide new information that isn’t available elsewhere in many different formats. However, some examples are very interesting and could merit more space. These are: Example 2 about the cautionary tale – this is a new point that I have not heard before; Example 6 which gives a very interesting example of when qualitative research and involvement overlap (again, would merit more discussion); Example 9 which is very brief but points to how protectionist policies can exclude people from participating (a common reason for not involving children, young people, people with disabilities, the frail elderly and other vulnerable populations) – I don’t think this has been considered in-depth by policies intended to increase participation; Example 10 is good on details on *how *researchers can work collaboratively with patients/carers/members of the public and will be of interest to people looking for new involvement ideas.

 You describe patients as primarily fulfilling three functions when they are involved in research: research decision-making, enhancing the patient perspective, how to capture knowledge. I suspect many involved patients/public advisors will object to this as being too limiting. In my own work I have seen at least three additional kinds of input: 1) public advisors helping researchers plan involvement in their research, 2) public advisors helping with dissemination and collaboration strategies (recently a public partner pointed out that the researchers had not asked for a letter of support to a very key national charity which could really help with dissemination), and 3) what I’d call ‘hidden or obvious talents’. Examples of the latter are patients drawing on their previous careers or hobbies, or other talents for seeing new aspects of an established research method. A parent carer we work with decided to use VideoScribe to disseminate some research she had initiated, she then presented about this software at a research seminar, and the research programme bought the software as a direct result of hearing her talk about it. In this example the function of the involved parent was to influence the dissemination and communication strategy of a whole programme of work.

 I hope these comments are useful.

I have read this submission. I believe that I have an appropriate level of expertise to confirm that it is of an acceptable scientific standard, however I have significant reservations, as outlined above.

# Peer review: Referee response for version 1

**DOI:** 10.5256/f1000research.16517.r35144

**Published:** 2018-06-25

**Authors:** Gary Hickey

**Affiliations:** 1 INVOLVE, Southampton, UK; 2 National Institute for Health Research, London, UK

A helpful article.  A few recommendations that will help improve the accuracy and help avoid any confusion.

 One step that is missing I think is 'Preparing the research team for PPI'. Support and training are mentioned in relation to the public  but this also applies to the research team.

 p1.  'The research methods of PPI...'  I don't think 'methods' is the right word (eg 'informal discussions' is not a research method).  Perhaps 'techniques' or 'The ways in which patients and public are involved in research...'

 p3.  I think the sentence in the first para beginning 'In the UK, INVOLVE states...' should come at the end of that para.

 p3.  'Steps for how etc'.  Are these steps?  Or are they 'Issues to consider'?

 p3.  'Understand what patient and public involvement is'.  A more accurate heading would be 'Understand your rationale for patient and public involvement.'  And in the next two sentences you mention 'theory'. I don't think these are theories - I think it is about 'understanding the rationale or motivation for patient and public involvement.'

 p3 'How does patient involvement differ from patient participation?'  The only step that is posed as a question - I'd change to 'Understanding how patient involvement differs etc'.  And it would be worth adding in again to this section (I know it's already in elsewhere) INVOLVE's distinction between involvement and participation.

 p7 The list of acronyms includes both terminology and organisations.  Confusing.  Take out the organisations - if they're in the main body of the article then they should be written in full anyway.

 p11 'A first step....'.  I'd reword to make less like an instruction and consistent with the rest of the article.  So 'A researcher could search the internet etc.  Another approach would be to contact migraine charities etc.'

 p11 Para beginning 'Lay or professional coordinators or link people...'  Consider explaining what these terms mean.  And replace 'whereas' with 'and'.  Lose the sentence 'The qualitative research and PPI become synergistic' - it confuses the point being made in this section and I'm nort sure it's accurate.

 p11 'Decide who and how many patients to involve' - we've moved from people to patients.  Needs to be consistent throughout.  Also add in  something about why you might want to consider having more than one person ie a) public can support each other b) helps redress the power balance in the room and c) the public can not always make a meeting and so, if you have more than one person, it reduced the likelihood that the public voice will be absent at any given time.

 p11 Replace the sentence 'Aim to find patients who represent the demogaphics etc' with 'Consider the demographic of those affected etc'.  Some readers will take the first sentence to the extreme and it may become a barrier to involving people.  I would also suggest losing the sentence 'It can be challenging to access 'typical' members etc' - I'm not sure what is meant by 'typical' here.  Need to give some consideration here to the issues of 'representativeness' - when you have only one or two people involved in your research it is unlikely that can be 'the' voice of everyone but they can be 'a' voice.  If you want something more representative then surveys etc might be a more appropriate answer.

 p11 The authors say that 'the more confident and financially secure are more likely to volunteer'.  Need to add in something about researchers have struggled to access certain groups.  The sentence 'A lack of resources' - not sure that 'less privileged' is a phrase I would use .  Perhaps 'less well off' or something similar? 

 p11 Need to be careful with the sentence 'Yet this is important..' - some ethnic minority groups might be offended that you are asserting that they tend' to experience lower health status' .  Perhaps 'some groups tend to experience etc'

 p12  'Patients can contribute to three key functions etc'.  Lose this sentence.  I'm not sure that it's true.  For example they can also be included in data collection (also applies to the penultimate bullet point in 'key messages' on page 15).

 p12  'AT an early stage a researcher is advised to discuss roles and task etc' - I would also add in here behaviours or responsibilities.

 p13  'INVOLVE has a Patient-Led-Research-Hub to support patients who want to puruse their own research.'  Are you referring to INVODirect?  This is more a list of organisations who support active PPI in research.

 p13  'Researchers wanting to study migraine etc'.  Add on to the end of the sentence 'this entails.'  (I read it at first as the patients doing aspects of the governance issues).

 p15 Either lose the final bullet point on the key messages -  I found this confusing - or add in something like 'to ensure you get the public view'.

I have read this submission. I believe that I have an appropriate level of expertise to confirm that it is of an acceptable scientific standard.

# Response

**Published:** 2018-06-25

**Authors:** Pat Hoddinott

**Affiliations:** Dr, UK

Thank you Gary for these very helpful suggestions, in particular the sentences where we could confuse the reader.   

Pat Hoddinott
